# Pan-cancer analysis of ASB3 and the potential clinical implications for immune microenvironment of glioblastoma multiforme

**DOI:** 10.3389/fimmu.2022.842524

**Published:** 2022-12-21

**Authors:** Long Mu, Zhibin Han, Shengkun Yu, Aowen Wang, Dongjiang Chen, Sijia Kong, Yifei Gu, Lin Xu, Axiang Liu, Ruohan Sun, Yu Long

**Affiliations:** ^1^ Department of Neurosurgery, The First Affiliated Hospital of Harbin Medical University, Harbin, China; ^2^ Division of Neuro-Oncology and Preston A. Wells, Jr. Center for Brain Tumor Therapy, Lillian S. Wells Department of Neurosurgery, University of Florida, Gainesville, FL, United States; ^3^ Obstetrics and Gynecology Department, Peking University Shenzhen Hospital, Shenzhen, China; ^4^ Department of Neurology, The First Affiliated Hospital of Harbin Medical University, Harbin, China

**Keywords:** glioblastoma multiforme, E3 ubiquitin ligases, ASB3, tumor microenvironment, Tregs

## Abstract

**Background:**

Ankyrin repeat and SOCS Box containing 3 (ASB3) is an E3 ubiquitin ligase. It has been reported to regulate the progression of some cancers, but no systematic pan-cancer analysis has been conducted to explore its function in prognosis and immune microenvironment.

**Method:**

In this study, mRNA expression data were downloaded from TCGA and GTEx database. Next generation sequencing data from 14 glioblastoma multiforme (GBM) samples by neurosurgical resection were used as validation dataset. Multiple bioinformatics methods (ssGSEA, Kaplan-Meier, Cox regression analysis, GSEA and online tools) were applied to explore ASB3 expression, gene activity, prognosis of patients in various cancers, and its correlation with clinical information, immune microenvironment and pertinent signal pathways in GBM. The biological function of ASB3 in tumor-infiltrating lymphocytes (TILs) was verified using an animal model.

**Results:**

We found that ASB3 was aberrant expressed in a variety of tumors, especially in GBM, and significantly correlated with the prognosis of cancer patients. The level of ASB3 was related to the TMB, MSI and immune cell infiltration in some cancer types. ASB3 had a negative association with immune infiltration and TME, including regulatory T cells (Tregs), cancer-associated fibroblasts, immunosuppressors and related signaling pathways in GBM. ASB3 overexpression reduced the proportion of Tregs in TILs. GSEA and PPI analysis also showed negative correlation between ASB3 expression and oncogenetic signaling pathways in GBM.

**Conclusion:**

A comprehensive pan-cancer analysis of ASB3 showed its potential function as a biomarker of cancer prognosis and effective prediction of immunotherapy response. This study not only enriches the understanding of the biological function of ASB3 in pan-cancer, especially in GBM immunity, but also provides a new reference for the personalized immunotherapy of GBM.

## Introduction

Cancer is the leading cause of death in every country in the world and an important obstacle to improving life expectancy, there is no absolute cure for cancer today (1, 2). Glioblastoma multiforme (GBM) is the most common malignant primary brain tumor with 49.1% of malignant brain tumors (3). Although the comprehensive treatment of GBM has made progress including surgery, radiotherapy and chemotherapy, the overall prognosis is still poor and the long-term survival rate remains low (4).

In recent years, cancer immunotherapy has become a prominent cancer treatment, especially immune checkpoint inhibitors (ICIs) therapy (5). Previous studies have shown that the disturbance of tumor microenvironment (TME), especially the tumor immune microenvironment (TIME), is one of the main causes of tumor malignant progression (6). However, many cancer patients do not achieve a persistent response to currently available antigens (7). The immune system in the brain follows different principles from the immune system elsewhere, not only is the access to the tumor restricted by the blood-brain barrier (BBB), but the host is subjected to considerable endogenous and therapeutically induced immunosuppression (8). Hence, exploring novel immunotherapeutic biomarkers, especially from GBM, can help cancer patients to formulate precise immunotherapy strategies and achieve more durable immune responses.

The ubiquitin-proteasome system (UPS) is one of the most important ways to regulate protein levels and a series of physiological and pathological processes in various organisms, controlling most proteins *via* ubiquitination and deubiquitination of substrates (9). The ankyrin repeat and suppressor of cytokine signaling (SOCS) box containing (ASB) family is comprised of 18 proteins and belongs to the SOCS box protein superfamily, they interact with Cul5-Rbx2 to form E3 ubiquitin ligases (E3s) and play important roles through ubiquitination-mediated pathway (10). The ASB3 gene from the ASB family is located on chromosome 2p16.2, which has 3 transcriptional variants encoding 2 isoforms. The ASB3 protein contains 518 amino acid residues, constituting 11 linked ankyrin repeats, followed by a SOCS box domain at the C-terminal of the peptide chain (11). ASB3 has been reported to play important roles in a variety of biological processes. In hepatocellular carcinoma (HCC), ASB3 knockdown promotes mitochondrial apoptosis by activating the interdependent cleavage of Beclin1 and caspase-8 (12). The mutations and down-regulated expression of ASB3 gene promote the growth and metastasis of colorectal cancer (CRC) cells (13). Furthermore, ASB3 mediates ubiquitination and degradation of tumor necrosis factor receptor II (TNFR2) (14). At present, there are few systematic studies on ASB3 in pan-cancer, especially in GBM.

In this study, we applied multiple bioinformatics methods to explore the expression level and gene activity of ASB3 in pan-cancer. Meanwhile, the relationship between ASB3 and GBM TME and its related signaling pathways was comprehensively analyzed in combination with public databases and sequencing data of patients in our institution. The role of ASB3 expression in the TIME was biologically validated using an animal model. The results indicate that ASB3 may be a potential target for future cancer immunotherapy development.

## Materials and methods

### Clinical samples collection

During the period from 2019 to 2021, surgically resected samples from glioma patients were collected, and postoperative pathology showed GBM. With the consent of the patients or their families, partial samples were collected and stored in a -80°C refrigerator. This study has been approved by the Ethics Committee of the First Affiliated Hospital of Harbin Medical University, and all patients or their families consented. Samples were sent to Novogene (https://novogene.com/) for quality inspection and sequencing. The raw data has been deposited in NCBI’s Sequence Read Archive (SRA, PRJNA786896).

### Data source

The gene expression RNA-seq mRNA profiles of GTEx (Genotype-Tissue Expression) cohorts of brain and GDC TCGA (The Cancer Genome Atlas) cohorts of 33 cancer types, and their relevant clinical information were downloaded from UCSC Xena (https://xena.ucsc.edu/). The gene list of E3s and deubiquitinating enzymes (DUBs) were downloaded from iUUCD (The Integrated Annotations for Ubiquitin and Ubiquitin-like Conjugation Database, http://iuucd.biocuckoo.org/) (15, 16).

### Pan-cancer analysis

After removing 8 cancer types without normal tissues, the mRNA expression data from 25 cancer types (BRCA, BLCA, COAD, CESC, CHOL, ESCA, GBM, HNSC, KIRP, KICH, KIRC, LGG, LIHC, LUSC, LUAD, PRAD, PAAD, PCPG, READ, SARC, SKCM, STAD, THYM, THCA and UCEC) were used to analyze ASB3 expression in normal and tumor tissues by Limma with a cutoff (*P* < 0.05). The ssGSEA (single-sample Gene Set Enrichment Analysis) was used to explore the gene activity of ASB3 by the R package “GSVA”. Patients were divided into ASB3 high expression group and low expression group according to the median value of ASB3 expression. Kaplan-Meier (KM) curves and Cox regression were used for evaluating the effect of ASB3 mRNA expression on the survival time and prognosis of patients with cancer. The correlation between ASB3 expression and tumor mutational burden (TMB) or microsatellite instability (MSI) were calculated utilizing the Spearman’s rank correlation coefficient by the R package “fmsb”.

### TME and TIME analysis

Estimation of STromal and Immune cells in MAlignant Tumours using Expression data (ESTIMATE) is an evaluation method to infer immune cells, stromal cells, and tumor purity in tumor samples according to gene expression characteristics (17). We used the R package “estimate” to obtain the infiltration levels of immune cells and stromal cells from the expression profile of GBM, and applied the Spearman’s rank correlation coefficient to evaluate their correlation with the mRNA expression level of ASB3. TISIDB (http://cis.hku.hk/TISIDB/) is an online website tool integrating a variety of data types and public databases, which provides multiple interactive information between tumor and the immune system (18). Tumor Immune Estimation Resource (TIMER) is a comprehensive tool for analyzing immune infiltrates of various cancers in a systematic way (19). TISIDB and TIMER were used to investigate the connection between ASB3 and multiple cancer immune microenvironments in the aspects of immune cell infiltration and immunosuppressor.

### Gene set enrichment analysis (GSEA)

GSEA is a widely-used computational method, which can extract effective biological insights from a large number of gene expression data (20, 21). It was applied to explore the biological pathways related to the ASB3 by using mRNA expression data from TCGA and clinical samples from neurosurgical resection of GBM patients.

### Functional enrichment analysis and PPI network construction

To explore the potential function of ASB3 in GBM, GSEA and Gene Ontology (GO) or Kyoto Encyclopedia of Genes and Genomes (KEGG) analyses were applied to identify signaling pathways most correlated with ASB3 by using mRNA expression data from TCGA. The protein-protein interaction (PPI) network was established by Metascape using genes negatively associated with ASB3. Cytoscape is a network visualization software program, and the plug-in Molecular COmplex DEtection (MCODE) can identify the most critical modules.

### Murine glioma lines

Mice glioma cell line (GL261) was gifted from Dr. Anhua Wu (The First Hospital of China Medical University, Shenyang, China). Cells were incubated at 37°C in an incubator with a gas environment of 5% CO_2_, and the medium used was Dulbecco’s modified eagle medium (DMEM) (C11995500BT, Gibco) containing 10% fetal bovine serum (FB15015, Clark) and a 1% penicillin-streptomycin mixture (15140-122, Gibco). The ASB3 overexpression sequence (NM_023906.3) was constructed by Comate Bioscience Co. Ltd. The recombinant lentivirus was prepared by transfecting pLVX-IRES-ZsGreen1 Vector (PT4064-5, ClonTech), pSPAX2 and pMD2.G (Hunan Fenghui Biotechnology) into 293T cells. After the lentiviral transfection, GL261 cells were cultured in a single clone by limiting dilution.

### Real-time quantitative reverse transcription-PCR

Total RNA was isolated from GL261 cells using E.Z.N.A.^®^ Total RNA Kit I (R6834, Omega). Then, the RNA concentration was determined using a NanoDrop One Spectrophotometer (Thermo Fisher Scientific), and reverse transcription was performed to obtain cDNA (RR047A, Takara). Finally, the expression level of ASB3 was determined through RT-qPCR using the Applied Biosystems StepOne machine (Applied Biosystems) and SYBR Green (4913914001, Roche). Primers for β-actin and ASB3 were purchased from Comate Bioscience Co. Ltd., with the following sequences: β-actin-F:5’-GTGACGTTGACATCCGTAAAGA-3’; β-actin-R:5’-GCCGGACTCATCGTACTCC-3’; ASB3-F:5’-TTGAAGTATGGAGCCCAGTTA-3’; ASB3-R:5’-CCAGCAAGCAGGAGATGTG-3’. Relative quantification of the gene expression was carried out *via* the double delta Ct analysis.

### Western blotting

GL261 cells were lysed in RIPA buffer (P0013E, Beyotime) containing protease inhibitor (BL612A, Biosharp) and phosphatase inhibitor (AR1183, Boster) cocktail. Protein concentration was measured using a BCA assay (P0010S, Beyotime). Anti-ASB3 (AP16752a, Abcepta) was used as the primary antibody, anti-GAPDH (10494-1-AP, Proteintech) was used as the control, and IRDye^®^ 800CW Goat anti-Rabbit IgG (D10629-12, LI-COR) was used as the secondary antibody. Western blotting was detected automatically using the Odyssey Infrared Imaging System (LI-COR).

### Brain tumor mice models

All *in vivo* experiments were conducted in accordance with protocols approved by the Animal Experimental Center of First Affiliated Hospital of Harbin Medical University and followed guidelines for animal welfare. Female C57BL/6 mice (6- to 8-week-old) were purchased from Liaoning Changsheng biotechnology co., Ltd. Mice were anesthetized and 1 × 10^5^ GL261 or ASB3 overexpression GL261 tumor cells in a 5μL volume were intracranially injected at 2.0mm lateral to the bregma at a depth of 3.0mm below the dura mater with a sterile Hamilton syringe fitted with a 26-gauge needle. Humanitarian endpoints were reached when animals exhibited the following deficits: (i) reluctant to move, (ii) weight loss >20% body weight, (iii) hunched posture, and (iv) lethargy.

### Flow cytometry analysis

Tumor-infiltrating lymphocytes (TILs) were collected from 37%, and 70% of the interface of Percoll (17-0891-09, GE Pharmacia) gradient, centrifuged at 500 × g for 30 minutes and washed once. Flow cytometry was performed using NovoCyte 2070R (Agilent Technologies). The viability of TILs was determined using the Zombie Red™ Fixable Viability Kit (423109, BioLegend). Cell surface staining antibodies, anti-CD3 (145-2C11), anti-CD4 (GK1.5), anti-CD8 (53-6.7), anti-PD-1 (RMP1-30), were purchased from BioLegend. The level of FOXP3 (Forkhead Box P3) were determined by intracellular staining using the True-Nuclear™ Transcription Factor Buffer Set (BioLegend) and anti-FOXP3 (FJK-16s, eBioscience). Appropriate isotype controls were used. All FCS data were analyzed using FlowJo 10.3 software.

### Immunohistochemistry

Paraffin sections with a thickness of 4μm were dewaxed and hydration in xylene and concentration gradient ethanol. Antigen repair was performed in EDTA buffer and heated under microwave for 18 minutes. Blocking was done using 5% BSA to reduce nonspecific staining. ASB3 (bs-7736R, Bioss) primary antibody working solution was added dropwise to the slides and placed in a wet box overnight at 4°C. The following day, after treatment according to the instructions of SABC-AP kit (SA1052, Boster), the exposed ASB3 protein was labeled with 0.01% DAB chromogenic solution and the nuclei were stained with hematoxylin. The staining results were observed under a light microscope.

### Statistical analysis

R software (v4.0.5) was used to analyze statistics. Identification of differentially expressed genes was assessed by Limma package with Wilcox Test (*P* filter = 0.05, log_2_FC filter = 2). The relation between various clinical characteristics of GBM patients and ASB3 expression was detected using Wilcoxon or Kruskal-Wallis tests. *P* < 0.05 represented significance in statistics. Experimental data are presented as mean ± SEM. Mann-Whitney tests were used to determine significant differences between two groups. Statistical analyses were performed using GraphPad Prism 9 software. *P* values of less than 0.05 were considered significant (*, *P* < 0.05; **, *P* < 0.01; ***, *P* < 0.001).

## Results

### Pan-cancer analysis of ASB3 expression

**Figure 1:**
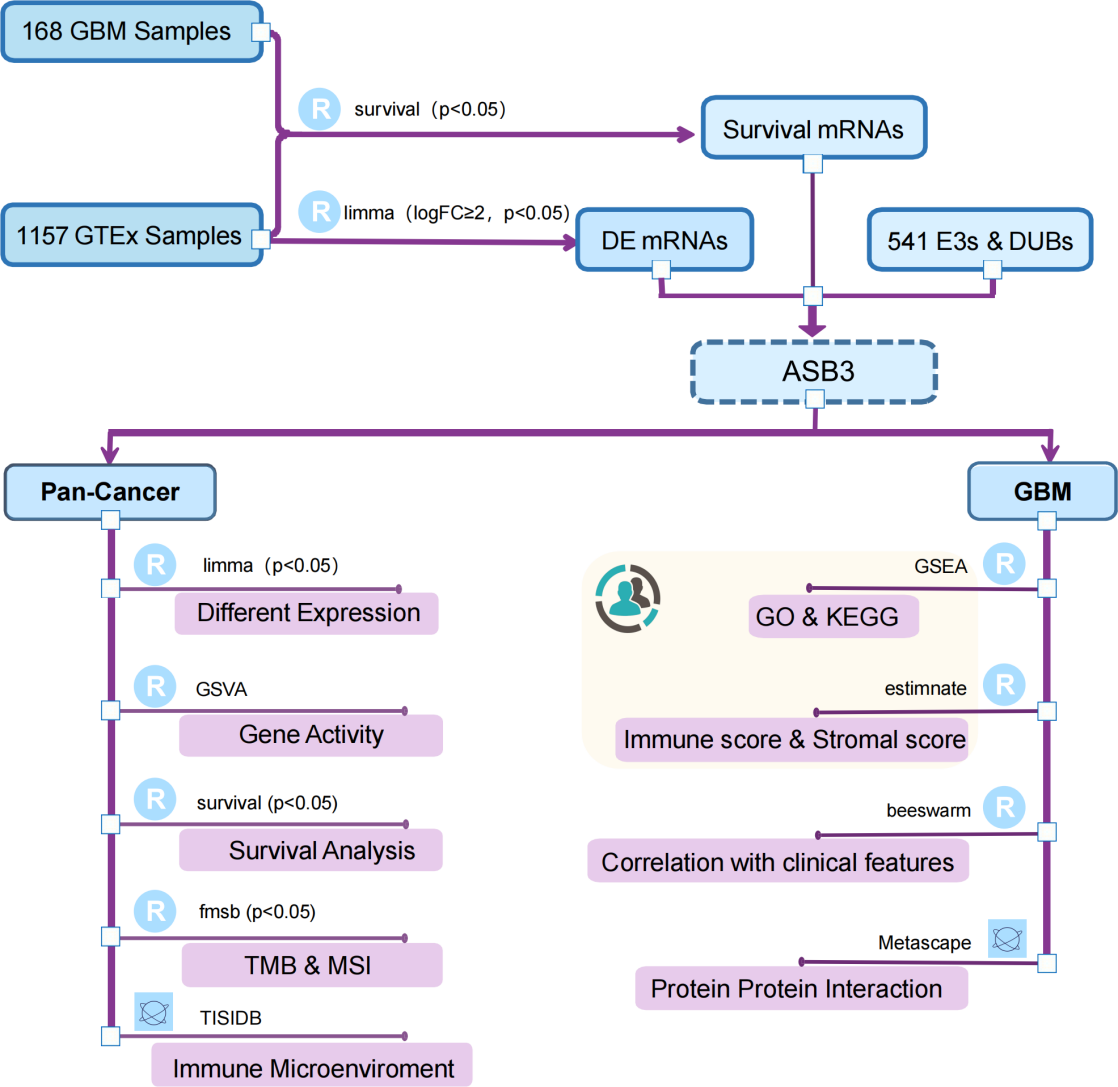
Flow chart of this study. The canary yellow background indicates an independent dataset validation from GBM patients of our institution.

**Figure 2:**
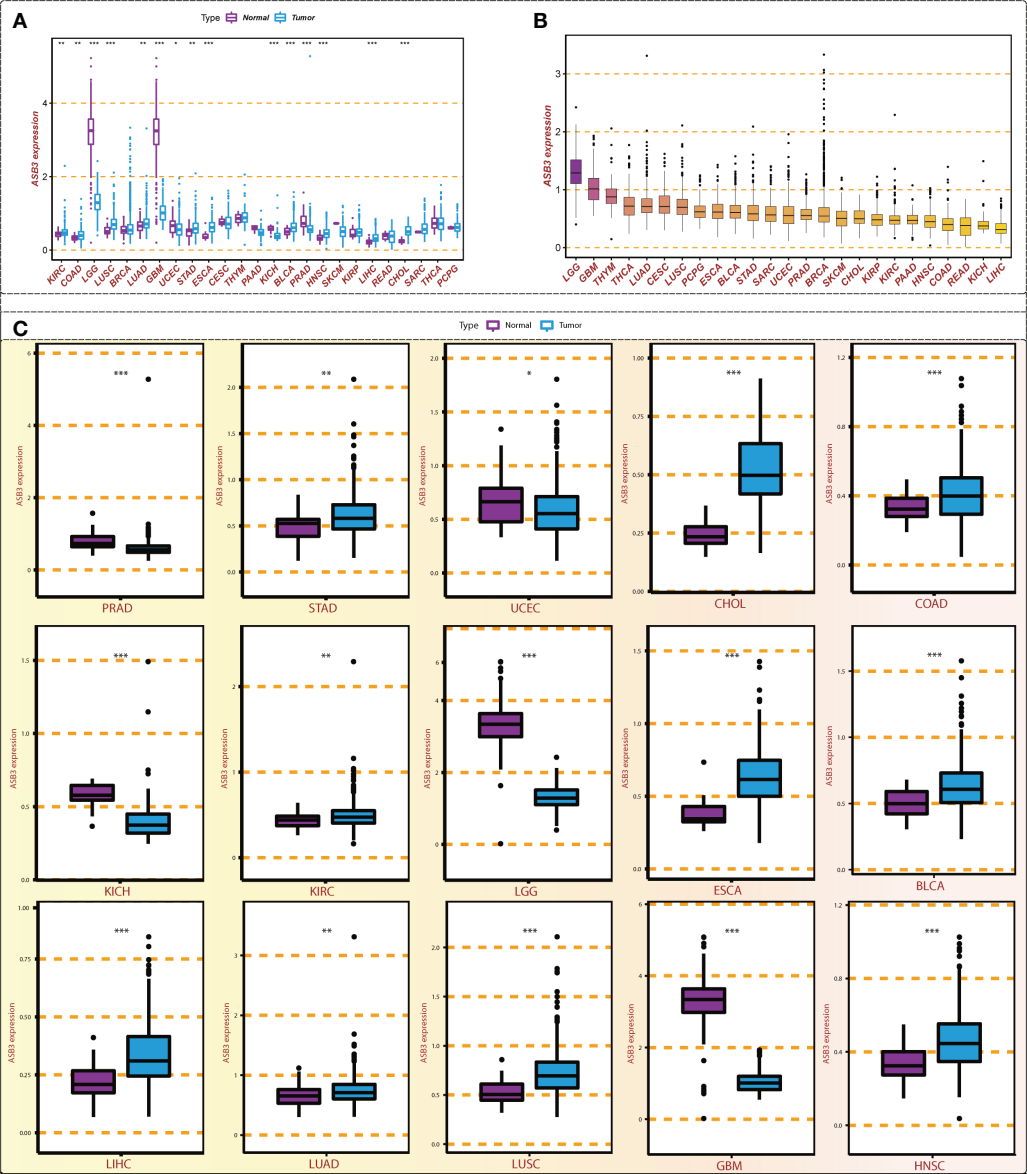
Expression level of ASB3 in pan-cancer. **(A)** ASB3 is abnormally expressed in different cancer types, up-regulated in 10 cancer types and down-regulated in 5 cancer types. **(B)** The level of ASB3 in pan-cancer, LGG and GBM have the highest level. **(C)** Box plots showing the significant different expression distribution of ASB3 across tumor and normal samples in 15 cancer types. *P < 0.05, **P < 0.01, ***P < 0.001.

Through the conjoint analysis of transcriptome data of normal brain tissues from GTEx and GBM samples from TCGA, we identified 1923 differentially expressed mRNAs and 149 mRNAs related to the survival of GBM patients. ASB3 was chosen to do the next analysis (**Figure 1**). In comparison with normal tissues, ASB3 mRNA expression was markedly upregulated in 10 types of tumors (BLCA, COAD, CHOL, ESCA, HNSC, KIRC, LUAD, LIHC, LUSC and STAD), and was notably downregulated in 5 cancer types (LGG, GBM, KICH, PRAD and UCEC, **Figure 2A**). We sorted the tumor types according to the gene expression levels of ASB3 (**Figure 2B**). Box plots demonstrated the significant different expression distribution of ASB3 across tumor and normal samples in 15 cancer types (**Figure 2C**). ASB3 was dysregulated in many kinds of cancers, particularly in GBM, illustrating ASB3 might have certain functions in GBM and other cancers.

### Pan-cancer analysis of ASB3 activity

**Figure 3:**
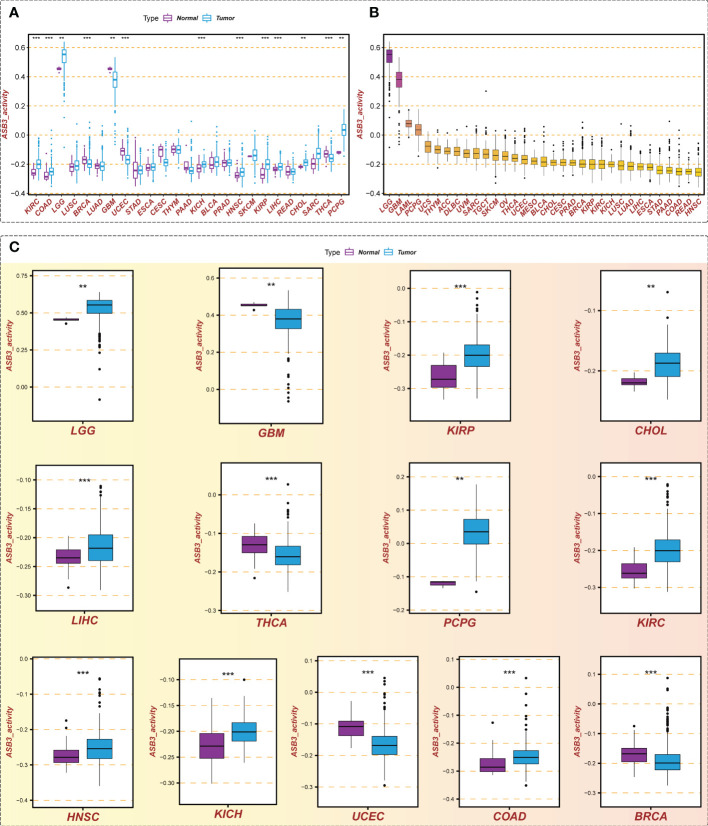
Gene activity of ASB3 in pan-cancer. **(A)** Comparison of ASB3 gene activity in a variety of cancers, increased in 9 cancer types and decreased in 4 cancer types. **(B)** The gene activity level of ASB3 in pan-cancer, LGG and GBM have the highest gene activity level. **(C)** Box plots showing the significant different activity of ASB3 across tumor and normal sample in 13 cancer types. **P < 0.01, ***P < 0.001.

We calculated the ASB3 gene activity of each sample by the ssGSEA algorithm and compared the ASB3 gene activity between normal and tumor groups. The results indicated that ASB3 gene activity was decreased in 4 cancer types including BRCA, GBM, THCA and UCEC, and was increased in 9 cancer types involving COAD, LGG, CHOL, LIHC, KIRP, KICH, KIRC, HNSC and PCPG compared with normal group (**Figure 3A**). According to the ranking of ASB3 gene activity in 33 cancer types, LGG and GBM were found to have the highest gene activity (**Figure 3B**). Box plots showed the significant different activity distribution of ASB3 across normal and tumor samples in 13 cancer types (**Figure 3C**). Based on the above results, we could infer that ASB3 plays a crucial role in GBM.

### Pan-cancer survival analysis of ASB3 expression

**Figure 4:**
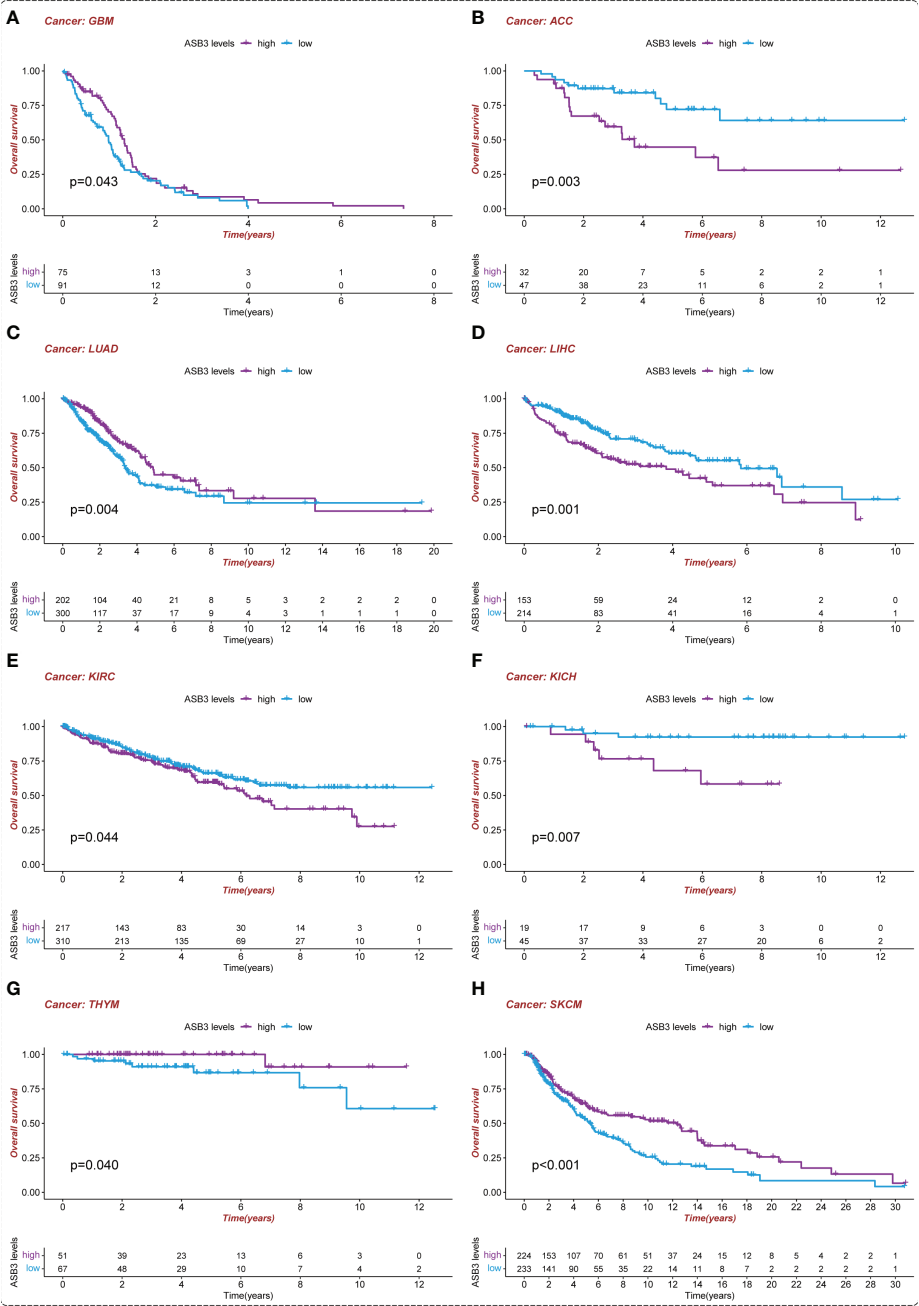
Kaplan-Meier survival analysis of ASB3 in pan-cancer. **(A)** The overall Kaplan-Meier survival analysis for ASB3 in GBM, **(B)** ACC, **(C)** LUAD, **(D)** LIHC, **(E)** KIRC, **(F)** KICH, **(G)** THYM and **(H)** SKCM.

To assess the prognostic value of ASB3, survival analysis was conducted in pan-cancer. We found that high-expressed ASB3 led to a worse prognosis in ACC, LIHC, KIRC and KICH. On the contrary, patients with high-expressed ASB3 had a better survival time in GBM, LUAD, SKCM and THYM (**Figure 4**). ASB3 may has diverse prognostic value in different cancers.

### Correlation of ASB3 and TMB or MSI in pan-cancer

By analyzing the relationship between ASB3 expression and TMB or MSI, we found that ASB3 was positively correlation with TMB in ACC, SKCM, MESO, LUSC and BLCA, conversely with TMB in THYM, PRAD and ESCA (**Figure S1A**). ASB3 was positively related with MSI in UCEC, THCA, SKCM, READ, LUSC, HNSC and COAD, negatively related with MSI in TGCT, KIRP, KIRC, GBM and CESC (**Figure S1B**). Thus, ASB3 may be used as the judgment index of TMB and MSI.

### Correlation of ASB3 expression with clinical characteristics of GBM

The TCGA RNA-seq data and clinical information were to explore the correlation between ASB3 expression and clinical features in GBM patients. The results shown that the expression level of ASB3 was lower in IDH wild-type patients (**Figure S2A**). ASB3 expression in classical and neural subtypes of GBM are higher than mesenchymal and proneural subtypes (**Figure S2B**). The expression of ASB3 is not related to radiotherapy, MGMT methylation, chemotherapy and age (**Figures S2C–F**).

### Correlation between ASB3 and immune microenvironment of GBM

**Figure 5:**
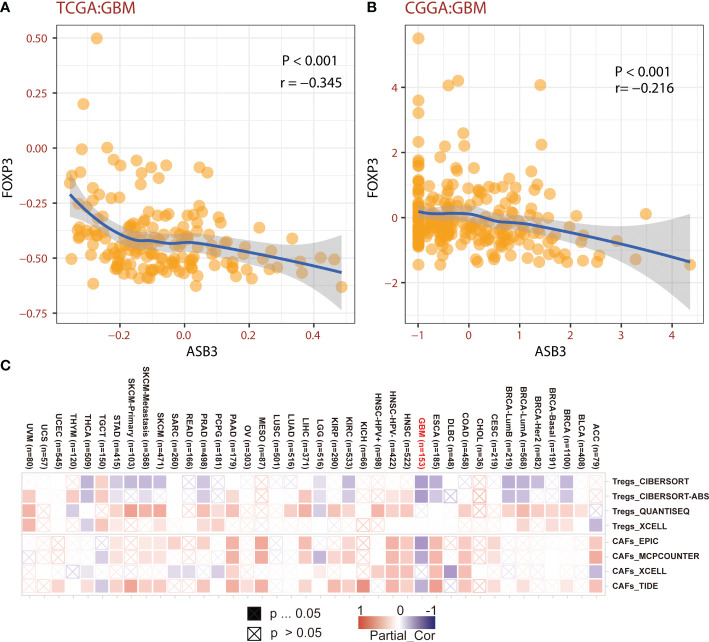
The correlation of ASB3 and Tregs. **(A)** Correlation coefficient of ASB3 and FOXP3 in TCGA GBM data. **(B)** Correlation coefficient of ASB3 and FOXP3 in CGGA GBM data. **(C)** Correlation heatmap of ASB3 and Tregs or CAFs in pan-cancer.

**Figure 6:**
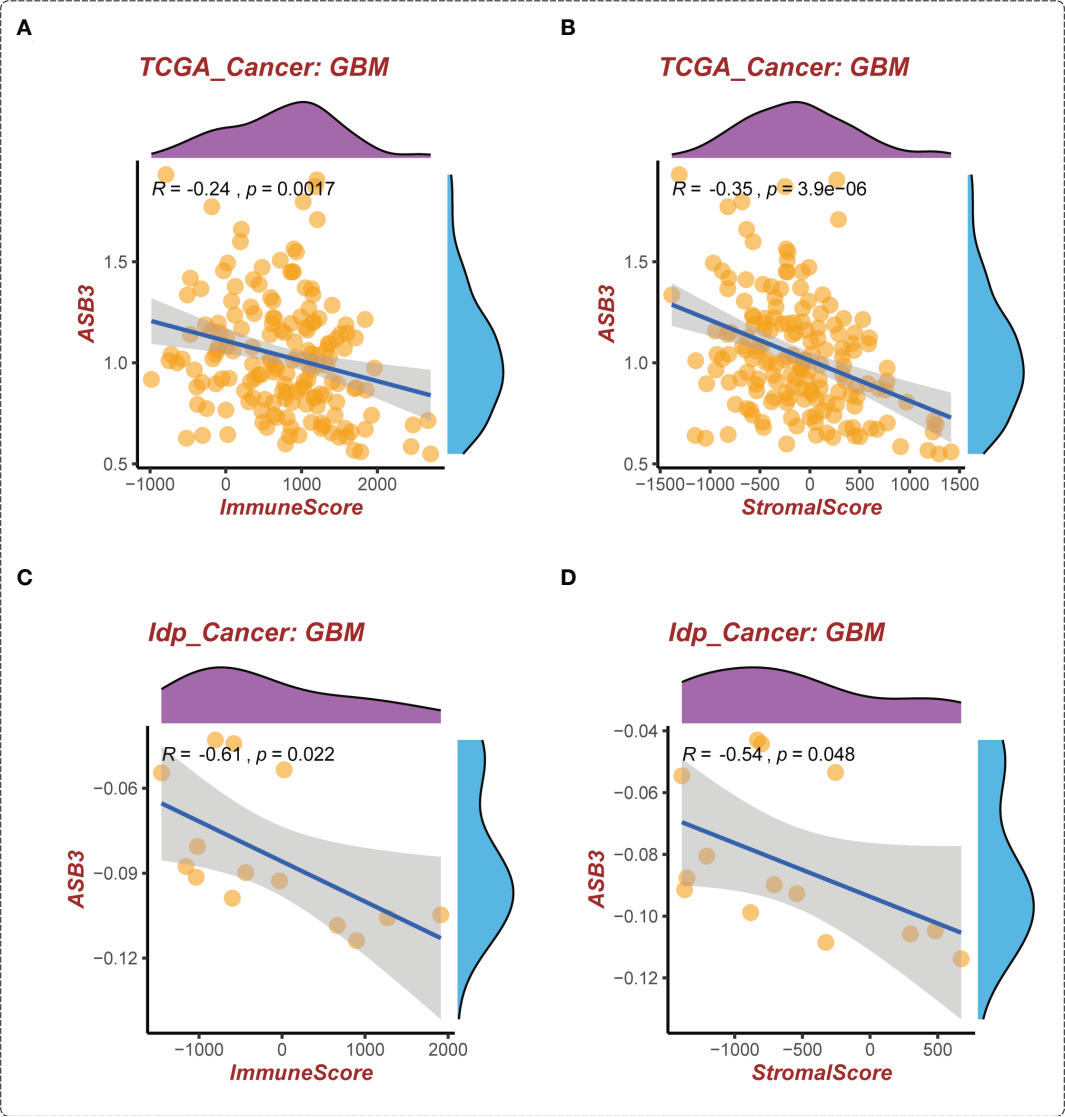
The correlation of ASB3 expression and TME in GBM. **(A)** Correlation between ASB3 and immune score in TCGA GBM data. **(B)** Correlation between ASB3 and stromal score in TCGA GBM data. **(C)** Correlation between ASB3 and immune score in independent GBM data. **(D)** Correlation between ASB3 and stromal score in independent GBM data.

The correlation between ASB3 and pan-cancer immune microenvironment was shown by heat map (**Figures S3A**, **S4A**). In GBM, ASB3 was positively related with effector memory T cells (TEM) (*r* = 0.24), and negatively related with regulatory T cells (Tregs) (*r* = -0.379) (**Figures S3B, C**). ASB3 was negatively correlated with immunosuppressors TGFB1 (Transforming Growth Factor Beta 1) (*r* = -0.411) and PD-1 (Programmed Cell Death 1) (*r* = -0.263) (**Figures S4B, C**). The expression of ASB3 was negatively correlated with FOXP3 both in TCGA (*r* = -0.345) and CGGA (*r* = -0.216) (**Figures 5A, B**). ASB3 level was significantly negatively associated with Tregs and cancer-associated fibroblasts (CAFs) (**Figure 5C**). More than that, the results of ESTIMATE analysis demonstrated ASB3 in GBM were negatively correlated with immune score (*r* = -0.24) and stromal score (*r* = -0.35) in TCGA (**Figures 6A, B**). With data from our own RNA-seq, ASB3 was also negatively correlated with immune score (*r* = -0.61) and stromal score (*r* = -0.54) (**Figures 6C, D**). We were surprised to find that renal cell carcinoma patients with high ASB3 expression were more responsive to anti-PD-1 targeted therapy (**Figure S7**).

### Functional enrichment analysis of ASB3 in GBM

**Figure 7:**
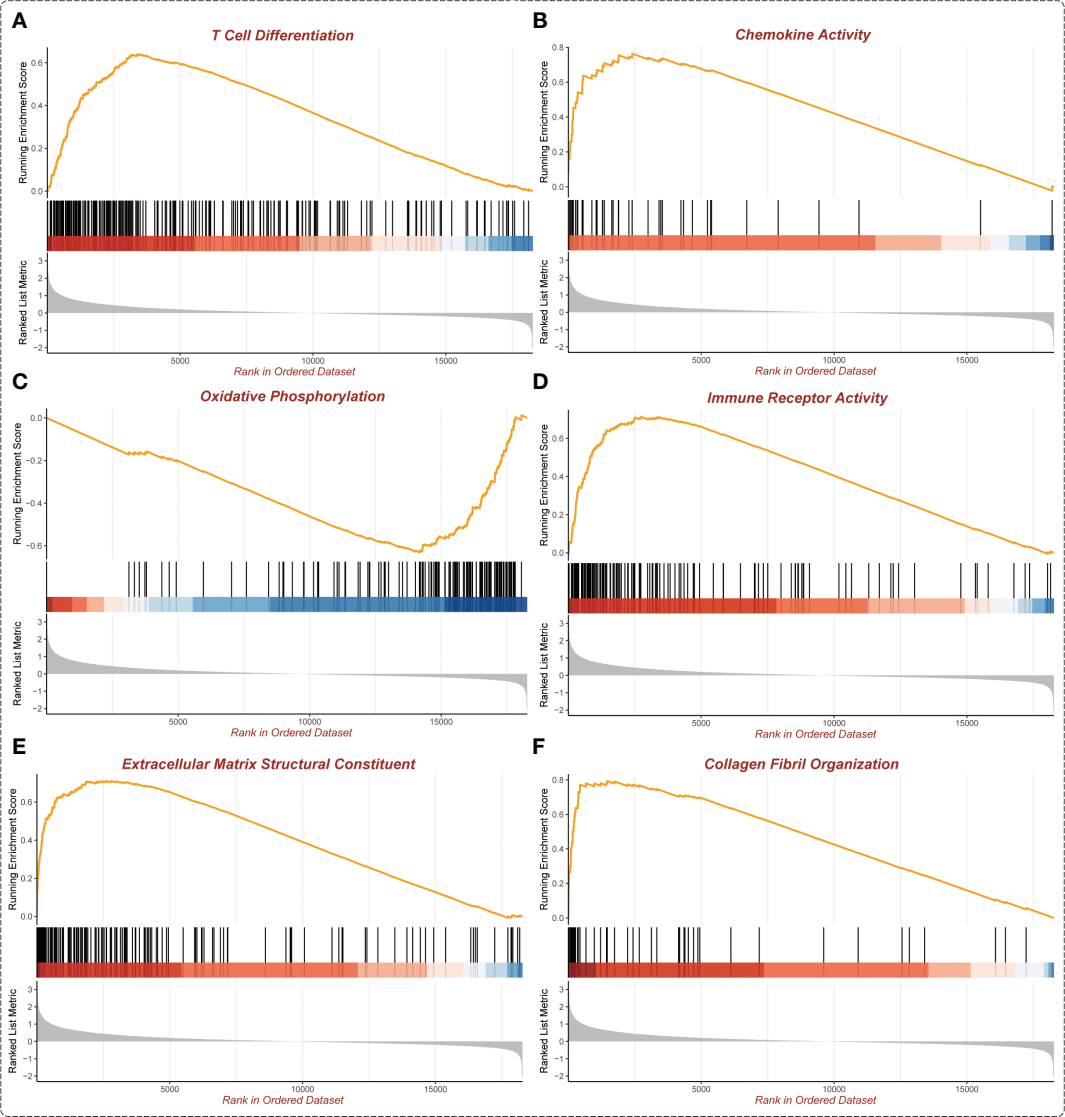
Enrichment plots from GSEA GO (TCGA). **(A)** T Cell Differentiation. **(B)** Chemokine Activity. **(C)** Oxidative Phosphorylation. **(D)** Immune Receptor Activity. **(E)** Extracellular Matrix Structural Constituent. **(F)** Collagen Fibril Organization.

**Figure 8:**
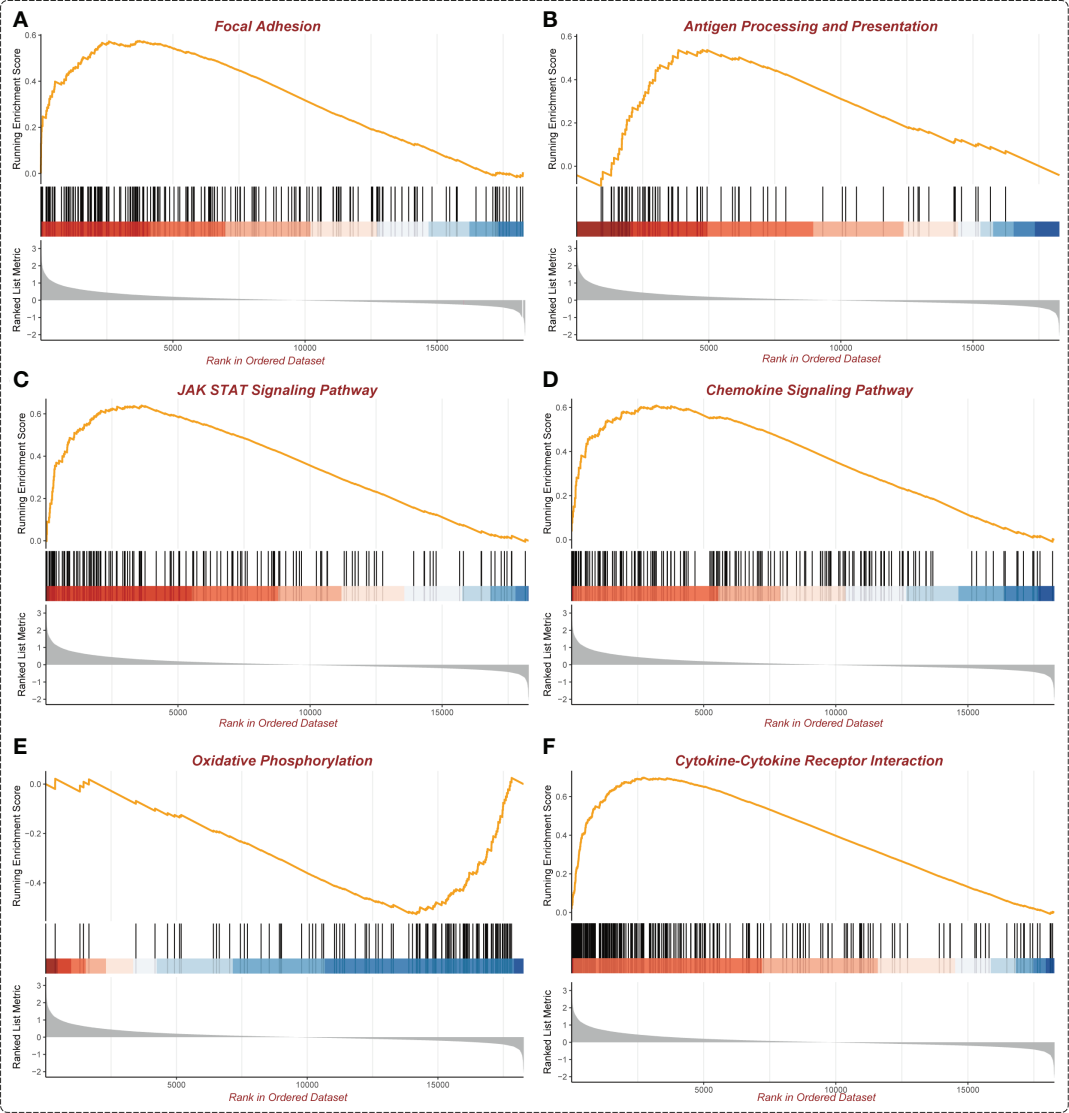
Enrichment plots from GSEA KEGG (TCGA). **(A)** Focal Adhesion. **(B)** Antigen Processing and Presentation. **(C)** JAK STAT Signaling Pathway. **(D)** Chemokine Signaling Pathway. **(E)** Oxidative Phosphorylation. **(F)** Cytokine-Cytokine Receptor interaction.

**Figure 9:**
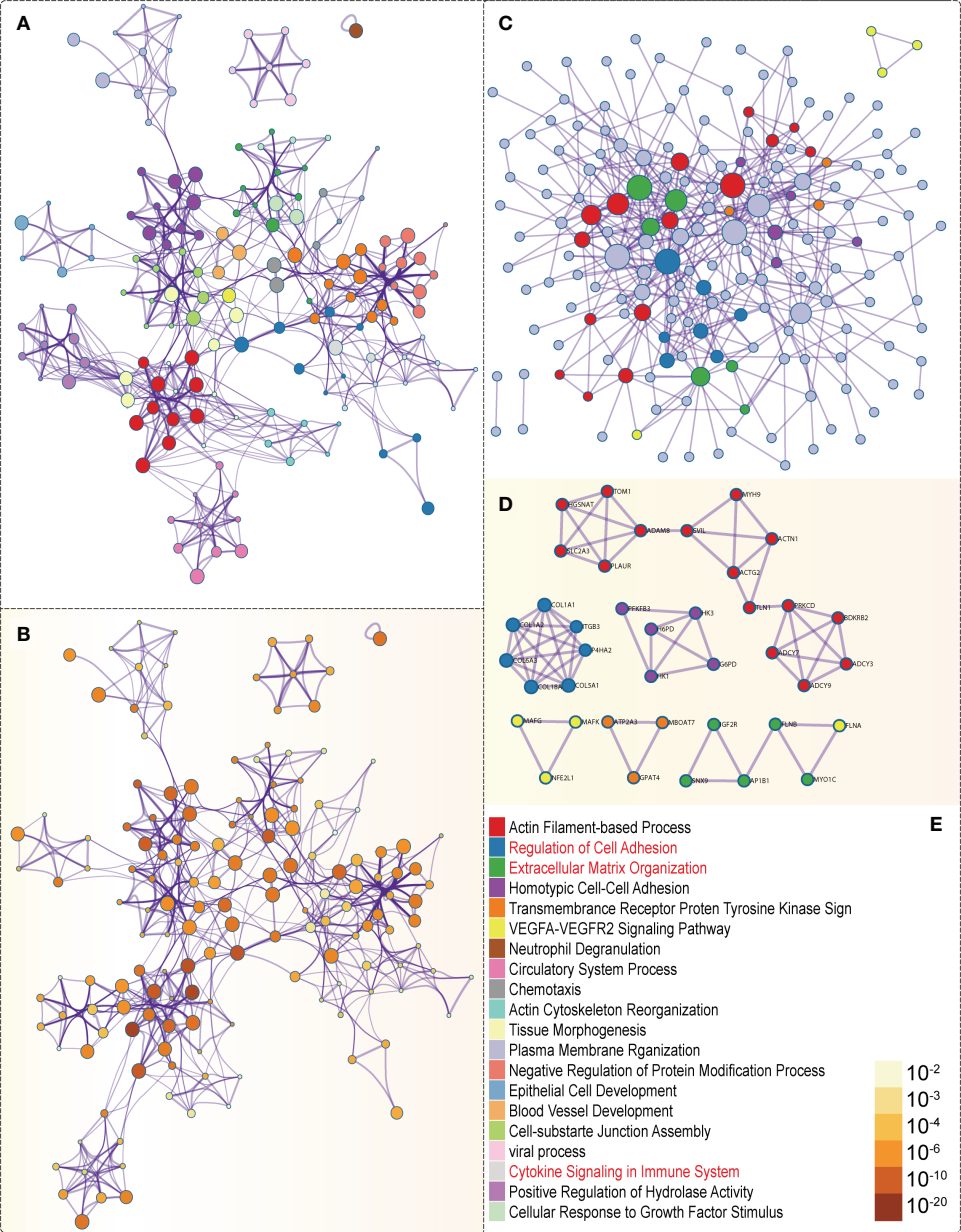
PPI network and mRNA enrichment terms negatively correlated with ASB3. **(A)** Colored by cluster ID. **(B)** Colored by P-value. **(C)** PPI network. **(D)** MCODE components. **(E)** Legend of **Figure 9**.

In the GSEA analysis of ASB3 related signaling pathways, we defined the ASB3 high expression group as ‘benign’ and the low expression group as ‘malignant’. The results showed that Oxidative Phosphorylation, Chemokine Activity, T Cell Differentiation, Immune Receptor Activity, Extracellular Matrix Structural Constituent and Collagen Fibril Organization were significantly enriched in GSEA GO terms (**Figure 7**). Focal Adhesion, Antigen Processing and Presentation, JAK STAT Signaling Pathway, Chemokine Signaling Pathway, Oxidative Phosphorylation and Cytokine-Cytokine Receptor Interaction were significantly enriched in GSEA KEGG terms (**Figure 8**). With data from our own RNA-seq, GSEA GO analysis demonstrated that B Cell Mediated Immunity, Cytokine Activity, Immunoglobulin Receptor Binding, Humoral Immune Response Mediated by Circulating Immunoglobulin, Antigen Binding and Complement Activation were significantly enriched (**Figure S5**). Toll Like Receptor Signaling Pathway, Lysosome, Chemokine Signaling Pathway, Cytokine-Cytokine Receptor Interaction, Cell Adhesion Molecules CAMS and Primary Immunodeficiency were significantly enriched in GSEA KEGG analysis (**Figure S6**). Gene functions such as Regulation of Cell Adhesion, Cytokine Signaling in Immune System and Extracellular Matrix Organization were identified by PPI (**Figures 9A, B**). PPI network and MCODE components designed by the gene lists were shown in **Figures 9C, D**.

### ASB3 overexpression reduced the proportion of Tregs *in vivo*

**Figure 10:**
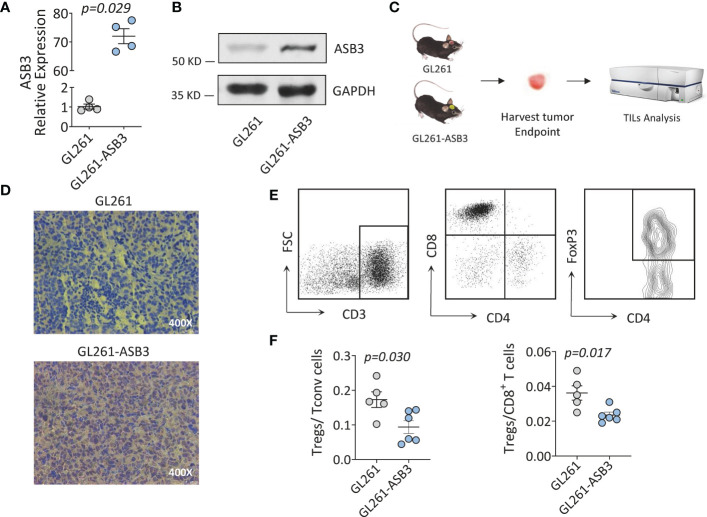
The Immunological functions of ASB3 *in vivo*. **(A)** ASB3 overexpression verification at mRNA level in GL261. **(B)** ASB3 overexpression verification at protein level in GL261. **(C)** Flow chart of mice experiments. Harvesting tumor derived TILs at the end of survival period for flow cytometry analysis. **(D)** ASB3 expression was detected by immunohistochemical in mice tumors at the end of survival. **(E)** Lymphocyte grouping in TILs. Phenotypical analysis was carried out by sequentially gating live cells, CD3^+^ T cells, CD8^+^ T cells, CD4^+^ T cells and FOXP3^+^ T cells. **(F)** The ratios of Tregs (CD3^+^ CD4^+^ FOXP3^+^)/Tconv (CD3^+^ CD4^+^ FOXP3^-^) and Tregs (CD3^+^ CD4^+^ FOXP3^+^)/CD8^+^ T cells in TILs.

We constructed ASB3 overexpression lentiviral plasmid to obtain stable ASB3 overexpression GL261 cell line. The overexpression was verified by RT-qPCR, western blotting and immunohistochemistry (**Figures 10A, B, D**). Orthotopic tumor models were established using normal GL261 cells and ASB3 overexpression GL261 cells, respectively. At the end of survival, tumors were analyzed by immunohistochemistry and TILs were harvested for flow cytometry (**Figure 10C**). We found that the Tregs/Tconv and Tregs/CD8^+^ ratios were significantly decreased in ASB3 overexpression cell model (**Figures 10E, F**). The negative correlation between ASB3 expression and Tregs was confirmed with *in vivo* experiments.

## Discussion

Targeting protein degradation has been proven to be a valid strategy for cancer treatment, several components of the UPS have shown to be potential anticancer targets (22). E3s are pivotal components in ubiquitination reactions because they strictly control substrate specificity and affinity (23). E3s are closely related to tumorigenesis owing to they regulate tumor suppressors and oncogenes (24). Therefore, the substrate specificity of E3s indicates that they are promising targets of anticancer drugs (25). The association between ASB family proteins and cancers has been reported previously. Inhibition of ASB4 in PLC and MHCC97-L HCC cells hindered cell migration and invasion, when ASB4 regulated by miR-200a was ectopically expressed in Hep3B HCC cells, the enhanced migration rate was measured (26). Transfection with hASB-8 cDNA truncation mutant lacking SOCS box could suppress the cell growth of SPC-A1 cells, which indicates that this gene might be related to the development of lung adenocarcinoma (27). ASB9 expression status was an independent prognostic factor for CRC overall survival in multivariate analysis and its siRNA transfected cells showed significant high invasiveness (28).

Previous research has shown that ASB3 is a biomarker for HCC and CRC (12, 13), but little is known about its role in other cancer types to date. In this study, we performed a comprehensive bioinformatics analysis using patient data from different databases to determine the functional role of ASB3 in a variety of cancers. Among the 25 cancer types containing normal tissues, the expression of ASB3 in 15 cancer types between normal tissues and tumors was statistically significant. We found that the expression trend of ASB3 in multiple tumors was inconsistent. This situation also widely exists in other genes, which may be related to tissue specificity and TME (29, 30). ASB3 has the highest gene expression and activity in both LGG and GBM in this analysis. The KM survival curve of GBM using TCGA data clearly demonstrated that low ASB3 expression was linked to poor prognosis. We also explored the effect of ASB3 on the GBM clinical features, and the results showed that the expression of ASB3 in GBM patients with IDH mutations was higher than those with IDH wild-type, while the IDH mutations were considered to be crucial for better determining the prognosis (31). Moreover, the level of ASB3 was also distinctness in different pathological subtypes of GBM. The above results are sufficient to illustrate that ASB3 was a potential prognostic biomarker in various cancers types, especially in GBM.

In recent years, cancer immunotherapy has shown prominent clinical effects by recovering the ability of the immune system to recognize and destruct tumor cells, but only for a few tumors (32). Tumor-infiltrating immune cells play important roles in the TME, which not only provide a suitable microenvironment for the survival of tumor cells, but also are connected to the regulation of tumor immune surveillance and the response of tumor treatment (33, 34). Due to the crucial role of the immune system in protecting and battling tumors, we concentrated on the effect of ASB3 on immunity. TMB is a promising pan-cancer immunotherapeutic predictive biomarker and can lead immunotherapy to the era of precision medicine (35, 36). MSI can predict sensitivity to ICIs therapy, especially PD-1/PD-L1 inhibitors (37). Our analysis showed that ASB3 expression was correlated with TMB in 8 cancer types and with MSI in 12 cancer types. The results indicated that ASB3 have a regulatory effect on tumor immunity of several cancer types. Consequently, ASB3 may be a potential biomarker to predict the response to immunotherapy in patients with malignant tumors.

GBM is a ‘cold’ tumor due to the low number of immunoregulatory cells and high levels of immunosuppressive cells, thus it is considered to be highly resistant to immunotherapy (38). Due to the particularity of TIME, the research of GBM immunotherapy has become a major topic. By the analysis of multiple databases, we found that ASB3 was negatively correlated with Tregs, CAFs, TGFB1 and PD-1 in GBM. These factors produce a marked effect on inhibiting anti-tumor immune response and promoting tumor genesis and development in the TME. Tregs are recognized immunosuppressive cells, which can hamper protective immunosurveillance of neoplasia and hinder the effective anti-tumor immune response of tumor hosts, thereby promoting the development and progression of tumors (39, 40). Animal experiment results showed that ASB3 overexpression could reduce the proportion of Tregs in TILs. The mechanism may be related to the inhibition of TNFR2, which needs further study (14, 41). CAFs are a stromal cell population and the most important parts of the TME, activated CAFs can promote tumor growth, angiogenesis, invasion and metastasis, along with extracellular matrix (ECM) remodeling and even chemical resistance (42, 43). TGFB1 can suppress the generation, differentiation and function of effector T cells, induce Tregs to enter the TME and prevent the maturation of Dendritic cells (DCs) (44, 45). Antibodies targeting PD-1 or its ligand PD-L1 rescue T cells from exhausted status and restore immune response against cancer cells (46, 47). Ubiquitination and deubiquitination of PD-1/PD-L1 play important roles in the regulation of PD-1/PD-L1 protein stability and dynamics (48). To sum up, it can be inferred that ASB3 can be used as a potential indicator for GBM immunotherapy, which is helpful for individualized treatment.

The GSEA results suggested that ASB3 was closely linked to many important cancer and immune signaling pathways, mainly including cytokine, chemokine, antigen processing and presentation, focal adhesion and oxidative phosphorylation. The complex and dynamic interactions of the secreted cytokines, chemokines, growth factors, and their receptors mediate chronic inflammation and immunosuppressive TME, which contributes to the progression, metastasis and reduced response to treatment of tumors include GBM (49, 50). Antigen processing and presentation is a complex process that can be used by tumors to escape immune recognition. These mechanisms include the regulation of antigen expression, the surface level of HLA-I, and the changes of antigen processing and presentation mechanism in tumor cells (51, 52). Focal adhesion is a multifunctional organelle that mediates cell ECM adhesion, cytoskeleton regulation and signal transduction (53). Focal adhesion kinase (FAK) is a protein that mainly regulates adhesion signal transduction and cell migration. Researchers found that the activity of FAK was elevated in PDAC, and related with low CD8^+^ T cell infiltration and high fibrosis (54, 55). Previous study has found that HIF-1α acts as a metabolic switch for Tregs between glycolytic-driven migration and oxidative phosphorylation-driven immunosuppression in GBM (56). Our study discovered that the level of oxidative phosphorylation in ‘malignant’ group decreased. This implies that the effect of ASB3 on immune microenvironment might be *via* regulating metabolism.

In summary, our results indicated that ASB3 was aberrantly expressed in various cancers and significantly correlated with the prognosis of cancer patients. For different cancers, ASB3 expression will bring different prognosis outcomes, which is necessary to further study the specific role of ASB3 in each cancer. The level of ASB3 was related to the TMB, MSI and immune cell infiltration in some cancer types. ASB3 expression showed closely connect with the infiltration of immune cells into the TME in GBM, and it had been preliminarily verified in the mice model. ASB3 was identified as a promising biomarker for the prognosis prediction of GBM, and it could provide a reference for the realization of more precise and individualized immunotherapy in the future.

## Data availability statement

The datasets presented in this study can be found in online repositories. The names of the repository/repositories and accession number(s) can be found in the article/**Supplementary Material**.

## Ethics statement

The studies involving human participants were reviewed and approved by the Ethics Committee of the First Affiliated Hospital of Harbin Medical University. The patients/participants provided their written informed consent to participate in this study. Written informed consent was obtained from the individual(s) for the publication of any potentially identifiable images or data included in this article.

## Author contributions

ZH and SY jointly performed the analysis of the bioinformatics section. LM and AW collected the glioma samples and performed the cell and animal experiments. DC participated in the design of the follow-up experimental scheme and language correction of the article. SK, YG, LX and AL took part in data analysis and figure drawing. YL and RS designed the experiment and participated in all parts of this study. All authors contributed to the article and approved the submitted version.

## Supplementary material

Supplementary Figure 1 The correlation of ASB3 expression and TMB or MSI in pan-cancer. **(A)** Correlation coefficient between ASB3 expression and TMB, positively in 5 cancer types and negatively in 3 cancer types. **(B)** Correlation coefficient between ASB3 expression and MSI, positively in 7 cancer types and negatively in 5 cancer types. **P* < 0.05, ***P* < 0.01, ****P* < 0.001.

Supplementary Figure 2 The correlation analysis between ASB3 expression and clinical features of GBM. **(A)** IDH mutation status. **(B)** Subtypes of GBM. **(C)** Radiotherapy status. **(D)** MGMT promoter methylation status. **(E)** Chemotherapy status. **(F)** Age.

Supplementary Figure 3 The correlation of ASB3 expression and immune cell infiltration. **(A)** Correlation heatmap between ASB3 and immune cell infiltration in pan-cancer. **(B)** Spearman correlation test of ASB3 expression and TEM in GBM. **(C)** Spearman correlation test of ASB3 expression and Tregs in GBM.

Supplementary Figure 4 The correlation of ASB3 expression and immunosuppressors. **(A)** Correlation heatmap between ASB3 and immunosuppressors in pan-cancer. **(B)** Spearman correlation test of ASB3 expression and TGFB1 in GBM. **(C)** Spearman correlation test of ASB3 expression and PD-1 in GBM.

Supplementary Figure 5 Enrichment plots from GSEA GO (Independent cohort). **(A)** B Cell Mediated Immunity. **(B)** Cytokine Activity. **(C)** Immunoglobulin Receptor Binding. **(D)** Humoral Immune Response Mediated by Circulating Immunoglobulin. **(E)** Antigen Binding. **(F)** Complement Activation.

Supplementary Figure 6 Enrichment plots from GSEA KEGG (Independent cohort). **(A)** Toll Like Receptor Signaling Pathway. **(B)** Lysosome. **(C)** Chemokine Signaling Pathway. **(D)** Cytokine-Cytokine Receptor Interaction. **(E)** Cell Adhesion Molecules CAMS. **(F)** Primary Immunodeficiency.

Supplementary Figure 7 Correlation of ASB3 expression and immunotherapy response in human renal cell carcinoma.

## References

[1] Bray F Laversanne M Weiderpass E Soerjomataram I. The ever-increasing importance of cancer as a leading cause of premature death worldwide. Cancer (2021) 127(16):3029–30. doi: 10.1002/cncr.33587 34086348

[2] Siegel RL Miller KD Fuchs HE Jemal A. Cancer statistics, 2022. CA Cancer J Clin (2022) 72(1):7–33. doi: 10.3322/caac.21708 35020204

[3] Low JT Ostrom QT Cioffi G Neff C Waite KA Kruchko C. Primary brain and other central nervous system tumors in the united states (2014-2018): A summary of the cbtrus statistical report for clinicians. Neurooncol Pract (2022) 9(3):165–82. doi: 10.1093/nop/npac015 PMC9113389 35601966

[4] Tan AC Ashley DM Lopez GY Malinzak M Friedman HS Khasraw M. Management of glioblastoma: State of the art and future directions. CA Cancer J Clin (2020) 70(4):299–312. doi: 10.3322/caac.21613 32478924

[5] Ribas A Wolchok JD. Cancer immunotherapy using checkpoint blockade. Science (2018) 359(6382):1350–5. doi: 10.1126/science.aar4060 PMC7391259 29567705

[6] Li MO Wolf N Raulet DH Akkari L Pittet MJ Rodriguez PC. Innate immune cells in the tumor microenvironment. Cancer Cell (2021) 39(6):725–9. doi: 10.1016/j.ccell.2021.05.016 34129817

[7] Bagchi S Yuan R Engleman EG. Immune checkpoint inhibitors for the treatment of cancer: Clinical impact and mechanisms of response and resistance. Annu Rev Pathol (2021) 16:223–49. doi: 10.1146/annurev-pathol-042020-042741 33197221

[8] Sampson JH Gunn MD Fecci PE Ashley DM. Brain immunology and immunotherapy in brain tumours. Nat Rev Cancer (2020) 20(1):12–25. doi: 10.1038/s41568-019-0224-7 31806885 PMC7327710

[9] Jin WL Mao XY Qiu GZ. Targeting deubiquitinating enzymes in glioblastoma multiforme: Expectations and challenges. Med Res Rev (2017) 37(3):627–61. doi: 10.1002/med.21421 27775833

[10] Kohroki J Nishiyama T Nakamura T Masuho Y. Asb proteins interact with Cullin5 and Rbx2 to form E3 ubiquitin ligase complexes. FEBS Lett (2005) 579(30):6796–802. doi: 10.1016/j.febslet.2005.11.016 16325183

[11] Kile BT Viney EM Willson TA Brodnicki TC Cancilla MR Herlihy AS. Cloning and characterization of the genes encoding the ankyrin repeat and socs box-containing proteins asb-1, asb-2, asb-3 and asb-4. Gene (2000) 258(1-2):31–41. doi: 10.1016/s0378-1119(00)00402-9 11111040

[12] Zhang W Liu F Che Z Wu M Tang Z Liu J. Asb3 knockdown promotes mitochondrial apoptosis *via* activating the interdependent cleavage of Beclin1 and caspase-8 in hepatocellular carcinoma. Sci China Life Sci (2019) 62(12):1692–702. doi: 10.1007/s11427-018-9505-0 31016535

[13] Du WY Lu ZH Ye W Fu X Zhou Y Kuang CM. The loss-of-Function mutations and down-regulated expression of Asb3 gene promote the growth and metastasis of colorectal cancer cells. Chin J Cancer (2017) 36(1):11. doi: 10.1186/s40880-017-0180-0 28088228 PMC5237493

[14] Chung AS Guan YJ Yuan ZL Albina JE Chin YE. Ankyrin repeat and socs box 3 (Asb3) mediates ubiquitination and degradation of tumor necrosis factor receptor ii. Mol Cell Biol (2005) 25(11):4716–26. doi: 10.1128/MCB.25.11.4716-4726.2005 PMC1140645 15899873

[15] Gao T Liu Z Wang Y Cheng H Yang Q Guo A. Uucd: A family-based database of ubiquitin and ubiquitin-like conjugation. Nucleic Acids Res (2013) 41(Database issue):D445–51. doi: 10.1093/nar/gks1103 PMC3531133 23172288

[16] Zhou J Xu Y Lin S Guo Y Deng W Zhang Y. Iuucd 2.0: An update with rich annotations for ubiquitin and ubiquitin-like conjugations. Nucleic Acids Res (2018) 46(D1):D447–D53. doi: 10.1093/nar/gkx1041 PMC5753239 29106644

[17] Yoshihara K Shahmoradgoli M Martinez E Vegesna R Kim H Torres-Garcia W. Inferring tumour purity and stromal and immune cell admixture from expression data. Nat Commun (2013) 4:2612. doi: 10.1038/ncomms3612 24113773 PMC3826632

[18] Ru B Wong CN Tong Y Zhong JY Zhong SSW Wu WC. Tisidb: An integrated repository portal for tumor-immune system interactions. Bioinformatics (2019) 35(20):4200–2. doi: 10.1093/bioinformatics/btz210 30903160

[19] Li T Fan J Wang B Traugh N Chen Q Liu JS. Timer: A web server for comprehensive analysis of tumor-infiltrating immune cells. Cancer Res (2017) 77(21):e108–e10. doi: 10.1158/0008-5472.CAN-17-0307 PMC6042652 29092952

[20] Subramanian A Tamayo P Mootha VK Mukherjee S Ebert BL Gillette MA. Gene set enrichment analysis: A knowledge-based approach for interpreting genome-wide expression profiles. Proc Natl Acad Sci U.S.A. (2005) 102(43):15545–50. doi: 10.1073/pnas.0506580102 PMC1239896 16199517

[21] Mootha VK Lindgren CM Eriksson KF Subramanian A Sihag S Lehar J. Pgc-1alpha-Responsive genes involved in oxidative phosphorylation are coordinately downregulated in human diabetes. Nat Genet (2003) 34(3):267–73. doi: 10.1038/ng1180 12808457

[22] Yang H Chen X Li K Cheaito H Yang Q Wu G. Repurposing old drugs as new inhibitors of the ubiquitin-proteasome pathway for cancer treatment. Semin Cancer Biol (2021) 68:105–22. doi: 10.1016/j.semcancer.2019.12.013 PMC7316602 31883910

[23] Zheng N Shabek N. Ubiquitin ligases: Structure, function, and regulation. Annu Rev Biochem (2017) 86:129–57. doi: 10.1146/annurev-biochem-060815-014922 28375744

[24] Senft D Qi J Ronai ZA. Ubiquitin ligases in oncogenic transformation and cancer therapy. Nat Rev Cancer (2018) 18(2):69–88. doi: 10.1038/nrc.2017.105 29242641 PMC6054770

[25] Wang D Ma L Wang B Liu J Wei W. E3 ubiquitin ligases in cancer and implications for therapies. Cancer Metastasis Rev (2017) 36(4):683–702. doi: 10.1007/s10555-017-9703-z 29043469

[26] Au V Tsang FH Man K Fan ST Poon RT Lee NP. Expression of ankyrin repeat and socs box containing 4 (Asb4) confers migration and invasion properties of hepatocellular carcinoma cells. Biosci Trends (2014) 8(2):101–10. doi: 10.5582/bst.8.101 24815387

[27] Liu Y Li J Zhang F Qin W Yao G He X. Molecular cloning and characterization of the human asb-8 gene encoding a novel member of ankyrin repeat and socs box containing protein family. Biochem Biophys Res Commun (2003) 300(4):972–9. doi: 10.1016/s0006-291x(02)02971-6 12559969

[28] Tokuoka M Miyoshi N Hitora T Mimori K Tanaka F Shibata K. Clinical significance of Asb9 in human colorectal cancer. Int J Oncol (2010) 37(5):1105–11. doi: 10.3892/ijo_00000762 20878058

[29] Lou W Wang W Chen J Wang S Huang Y. Ncrnas-mediated high expression of Sema3f correlates with poor prognosis and tumor immune infiltration of hepatocellular carcinoma. Mol Ther Nucleic Acids (2021) 24:845–55. doi: 10.1016/j.omtn.2021.03.014 PMC8121632 34026328

[30] Gobin E Bagwell K Wagner J Mysona D Sandirasegarane S Smith N. A pan-cancer perspective of matrix metalloproteases (Mmp) gene expression profile and their Diagnostic/Prognostic potential. BMC Cancer (2019) 19(1):581. doi: 10.1186/s12885-019-5768-0 31200666 PMC6567474

[31] D’Alessio A Proietti G Sica G Scicchitano BM. Pathological and molecular features of glioblastoma and its peritumoral tissue. Cancers (Basel) (2019) 11(4):469. doi: 10.3390/cancers11040469 30987226 PMC6521241

[32] Wang ZL Wang Z Li GZ Wang QW Bao ZS Zhang CB. Immune cytolytic activity is associated with genetic and clinical properties of glioma. Front Immunol (2019) 10:1756. doi: 10.3389/fimmu.2019.01756 31428092 PMC6688525

[33] Bader JE Voss K Rathmell JC. Targeting metabolism to improve the tumor microenvironment for cancer immunotherapy. Mol Cell (2020) 78(6):1019–33. doi: 10.1016/j.molcel.2020.05.034 PMC7339967 32559423

[34] Zeng D Li M Zhou R Zhang J Sun H Shi M. Tumor microenvironment characterization in gastric cancer identifies prognostic and immunotherapeutically relevant gene signatures. Cancer Immunol Res (2019) 7(5):737–50. doi: 10.1158/2326-6066.CIR-18-0436 30842092

[35] Fumet JD Truntzer C Yarchoan M Ghiringhelli F. Tumour mutational burden as a biomarker for immunotherapy: Current data and emerging concepts. Eur J Cancer (2020) 131:40–50. doi: 10.1016/j.ejca.2020.02.038 32278982 PMC9473693

[36] Steuer CE Ramalingam SS. Tumor mutation burden: Leading immunotherapy to the era of precision medicine? J Clin Oncol (2018) 36(7):631–2. doi: 10.1200/JCO.2017.76.8770 29337637

[37] Amato M Franco R Facchini G Addeo R Ciardiello F Berretta M. Microsatellite instability: From the implementation of the detection to a prognostic and predictive role in cancers. Int J Mol Sci (2022) 23(15):8726. doi: 10.3390/ijms23158726 35955855 PMC9369169

[38] Salemizadeh Parizi M Salemizadeh Parizi F Abdolhosseini S Vanaei S Manzouri A Ebrahimzadeh F. Myeloid-derived suppressor cells (Mdscs) in brain cancer: Challenges and therapeutic strategies. Inflammopharmacology (2021) 29(6):1613–24. doi: 10.1007/s10787-021-00878-9 34613567

[39] Togashi Y Shitara K Nishikawa H. Regulatory T cells in cancer immunosuppression - implications for anticancer therapy. Nat Rev Clin Oncol (2019) 16(6):356–71. doi: 10.1038/s41571-019-0175-7 30705439

[40] Tanaka A Sakaguchi S. Regulatory T cells in cancer immunotherapy. Cell Res (2017) 27(1):109–18. doi: 10.1038/cr.2016.151 PMC5223231 27995907

[41] Moatti A Debesset A Pilon C Beldi-Ferchiou A Leclerc M Redjoul R. Tnfr2 blockade of regulatory T cells unleashes an antitumor immune response after hematopoietic stem-cell transplantation. J Immunother Cancer (2022) 10(4):e003508. doi: 10.1136/jitc-2021-003508 35387779 PMC8987798

[42] Mao X Xu J Wang W Liang C Hua J Liu J. Crosstalk between cancer-associated fibroblasts and immune cells in the tumor microenvironment: New findings and future perspectives. Mol Cancer (2021) 20(1):131. doi: 10.1186/s12943-021-01428-1 34635121 PMC8504100

[43] Chen X Song E. Turning foes to friends: Targeting cancer-associated fibroblasts. Nat Rev Drug Discovery (2019) 18(2):99–115. doi: 10.1038/s41573-018-0004-1 30470818

[44] Woo SR Corrales L Gajewski TF. Innate immune recognition of cancer. Annu Rev Immunol (2015) 33:445–74. doi: 10.1146/annurev-immunol-032414-112043 25622193

[45] de Streel G Lucas S. Targeting immunosuppression by tgf-Beta1 for cancer immunotherapy. Biochem Pharmacol (2021) 192:114697. doi: 10.1016/j.bcp.2021.114697 34302795 PMC8484859

[46] Yi M Zheng X Niu M Zhu S Ge H Wu K. Combination strategies with pd-1/Pd-L1 blockade: Current advances and future directions. Mol Cancer (2022) 21(1):28. doi: 10.1186/s12943-021-01489-2 35062949 PMC8780712

[47] Zhang H Dai Z Wu W Wang Z Zhang N Zhang L. Regulatory mechanisms of immune checkpoints pd-L1 and ctla-4 in cancer. J Exp Clin Cancer Res (2021) 40(1):184. doi: 10.1186/s13046-021-01987-7 34088360 PMC8178863

[48] Hu X Wang J Chu M Liu Y Wang ZW Zhu X. Emerging role of ubiquitination in the regulation of pd-1/Pd-L1 in cancer immunotherapy. Mol Ther (2021) 29(3):908–19. doi: 10.1016/j.ymthe.2020.12.032 PMC7934629 33388422

[49] Bhat AA Nisar S Maacha S Carneiro-Lobo TC Akhtar S Siveen KS. Cytokine-chemokine network driven metastasis in esophageal cancer; promising avenue for targeted therapy. Mol Cancer (2021) 20(1):2. doi: 10.1186/s12943-020-01294-3 33390169 PMC7780621

[50] Broekman ML Maas SLN Abels ER Mempel TR Krichevsky AM Breakefield XO. Multidimensional communication in the microenvirons of glioblastoma. Nat Rev Neurol (2018) 14(8):482–95. doi: 10.1038/s41582-018-0025-8 PMC6425928 29985475

[51] Jhunjhunwala S Hammer C Delamarre L. Antigen presentation in cancer: Insights into tumour immunogenicity and immune evasion. Nat Rev Cancer (2021) 21(5):298–312. doi: 10.1038/s41568-021-00339-z 33750922

[52] Lee MY Jeon JW Sievers C Allen CT. Antigen processing and presentation in cancer immunotherapy. J Immunother Cancer (2020) 8(2):e001111. doi: 10.1136/jitc-2020-001111 32859742 PMC7454179

[53] Kanchanawong P Shtengel G Pasapera AM Ramko EB Davidson MW Hess HF. Nanoscale architecture of integrin-based cell adhesions. Nature (2010) 468(7323):580–4. doi: 10.1038/nature09621 PMC3046339 21107430

[54] Dawson JC Serrels A Stupack DG Schlaepfer DD Frame MC. Targeting fak in anticancer combination therapies. Nat Rev Cancer (2021) 21(5):313–24. doi: 10.1038/s41568-021-00340-6 PMC8276817 33731845

[55] Jiang H Hegde S Knolhoff BL Zhu Y Herndon JM Meyer MA. Targeting focal adhesion kinase renders pancreatic cancers responsive to checkpoint immunotherapy. Nat Med (2016) 22(8):851–60. doi: 10.1038/nm.4123 PMC4935930 27376576

[56] Miska J Lee-Chang C Rashidi A Muroski ME Chang AL Lopez-Rosas A. Hif-1alpha is a metabolic switch between glycolytic-driven migration and oxidative phosphorylation-driven immunosuppression of tregs in glioblastoma. Cell Rep (2019) 27(1):226–37 e4. doi: 10.1016/j.celrep.2019.03.029 30943404 PMC6461402

