# Supplementary material for: Pan-cancer analysis of ASB3 and the potential clinical implications for immune microenvironment of glioblastoma multiforme
Source: Front Immunol. 2022 Dec 21;13:842524. doi: 10.3389/fimmu.2022.842524 (PMC9812557; doi:10.3389/fimmu.2022.842524)
Supplement: Supplementary file 1 [file DataSheet_1.docx]

https://dataview.ncbi.nlm.nih.gov/object/PRJNA786896?reviewer=5bh322evctch53sal3565appsq
