# Supplementary material for: Pan-cancer analysis of ASB3 and the potential clinical implications for immune microenvironment of glioblastoma multiforme
Source: Front Immunol. 2022 Dec 21;13:842524. doi: 10.3389/fimmu.2022.842524 (PMC9812557; doi:10.3389/fimmu.2022.842524)
Supplement: Supplementary file 2 [file DataSheet_2.docx]

https://www.jianguoyun.com/p/DUxa8LUQxMWUCxi71-YEIAA
