# Supplementary figures and images for: Pan-cancer analysis of ASB3 and the potential clinical implications for immune microenvironment of glioblastoma multiforme
Source: Front Immunol. 2022 Dec 21;13:842524. doi: 10.3389/fimmu.2022.842524 (PMC9812557; doi:10.3389/fimmu.2022.842524)

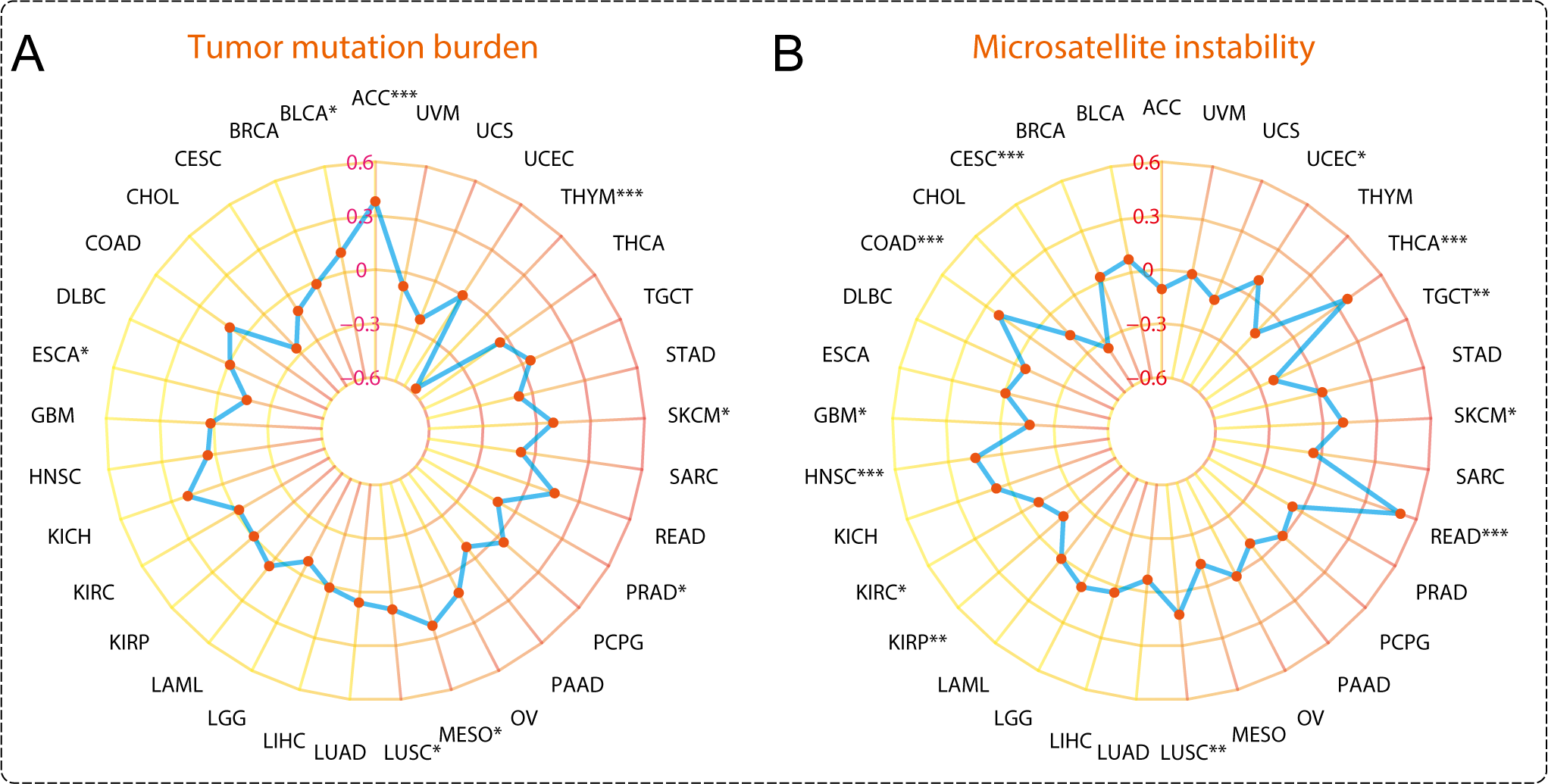

Supplement: Supplementary Figure 1 — The correlation of ASB3 expression and TMB or MSI in pan-cancer. (A) Correlation coefficient between ASB3 expression and TMB, positively in 5 cancer types and negatively in 3 cancer types. (B) Correlation coefficient between ASB3 expression and MSI, positively in 7 cancer types and negatively in 5 cancer types. *P < 0.05, **P < 0.01, ***P < 0.001. [file Image_1.tif]

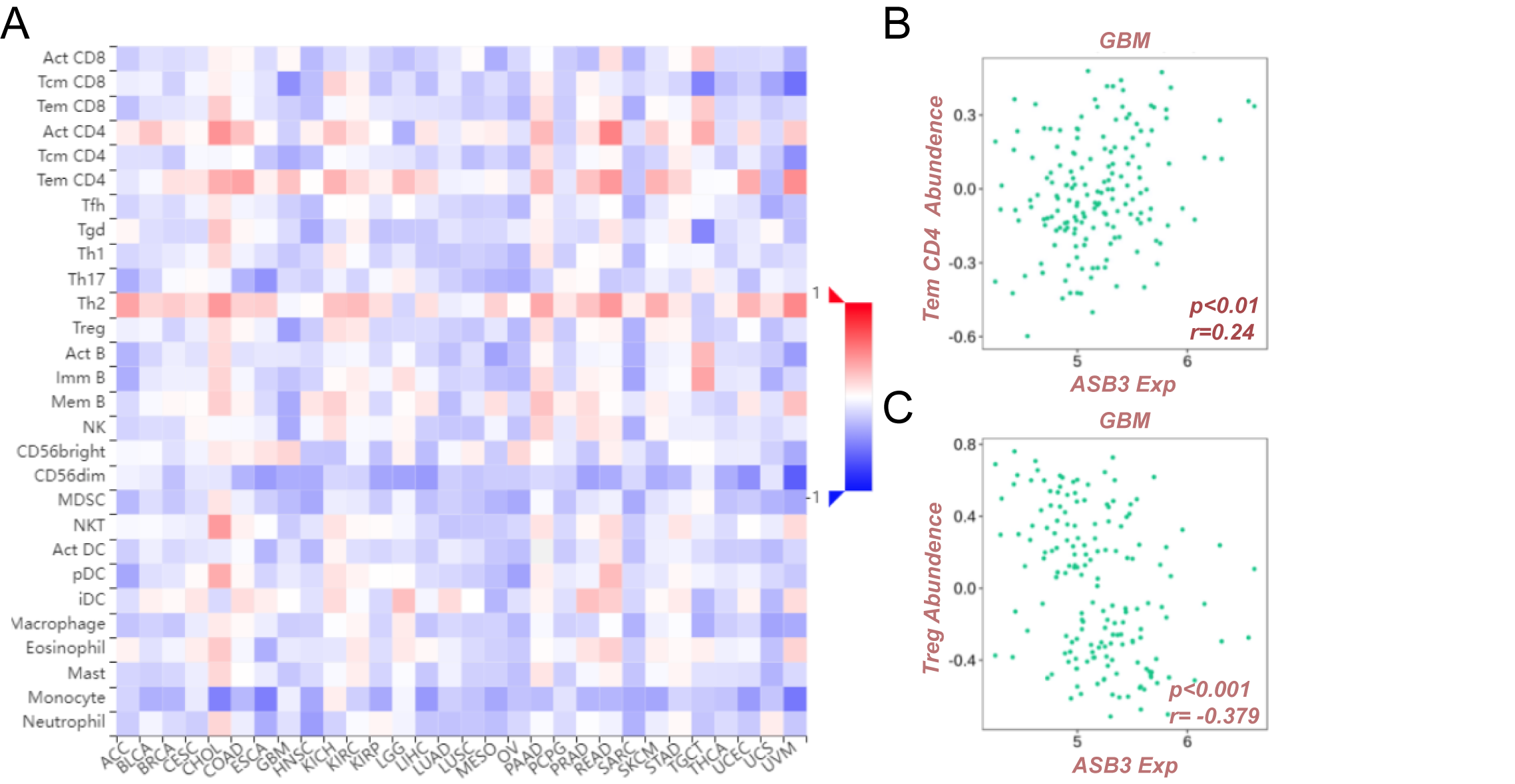

Supplement: Supplementary Figure 2 — The correlation analysis between ASB3 expression and clinical features of GBM. (A) IDH mutation status. (B) Subtypes of GBM. (C) Radiotherapy status. (D) MGMT promoter methylation status. (E) Chemotherapy status. (F) Age. [file Image_2.tif]

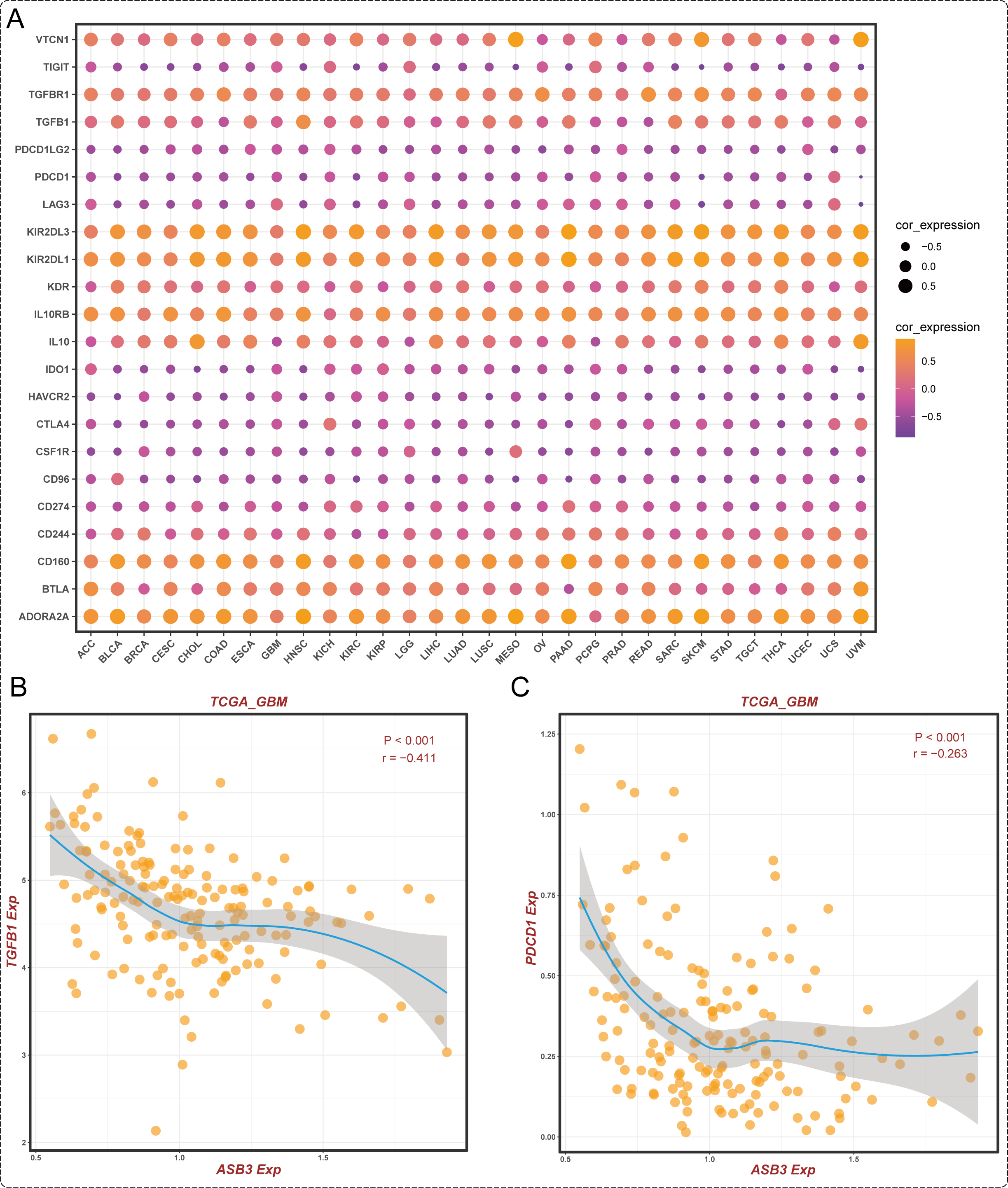

Supplement: Supplementary Figure 3 — The correlation of ASB3 expression and immune cell infiltration. (A) Correlation heatmap between ASB3 and immune cell infiltration in pan-cancer. (B) Spearman correlation test of ASB3 expression and TEM in GBM. (C) Spearman correlation test of ASB3 expression and Tregs in GBM. [file Image_3.tif]

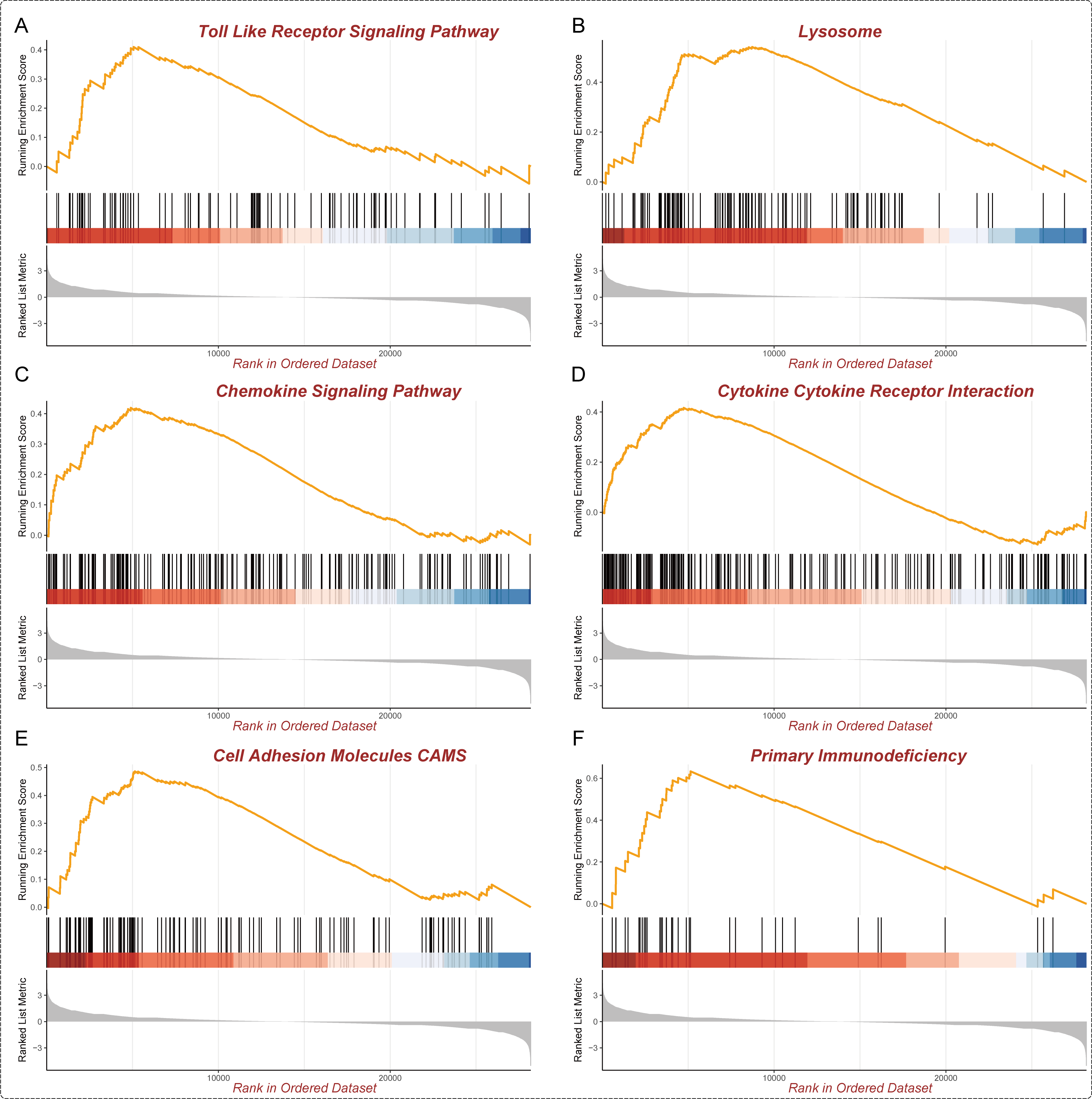

Supplement: Supplementary Figure 4 — The correlation of ASB3 expression and immunosuppressors. (A) Correlation heatmap between ASB3 and immunosuppressors in pan-cancer. (B) Spearman correlation test of ASB3 expression and TGFB1 in GBM. (C) Spearman correlation test of ASB3 expression and PD-1 in GBM. [file Image_4.tif]

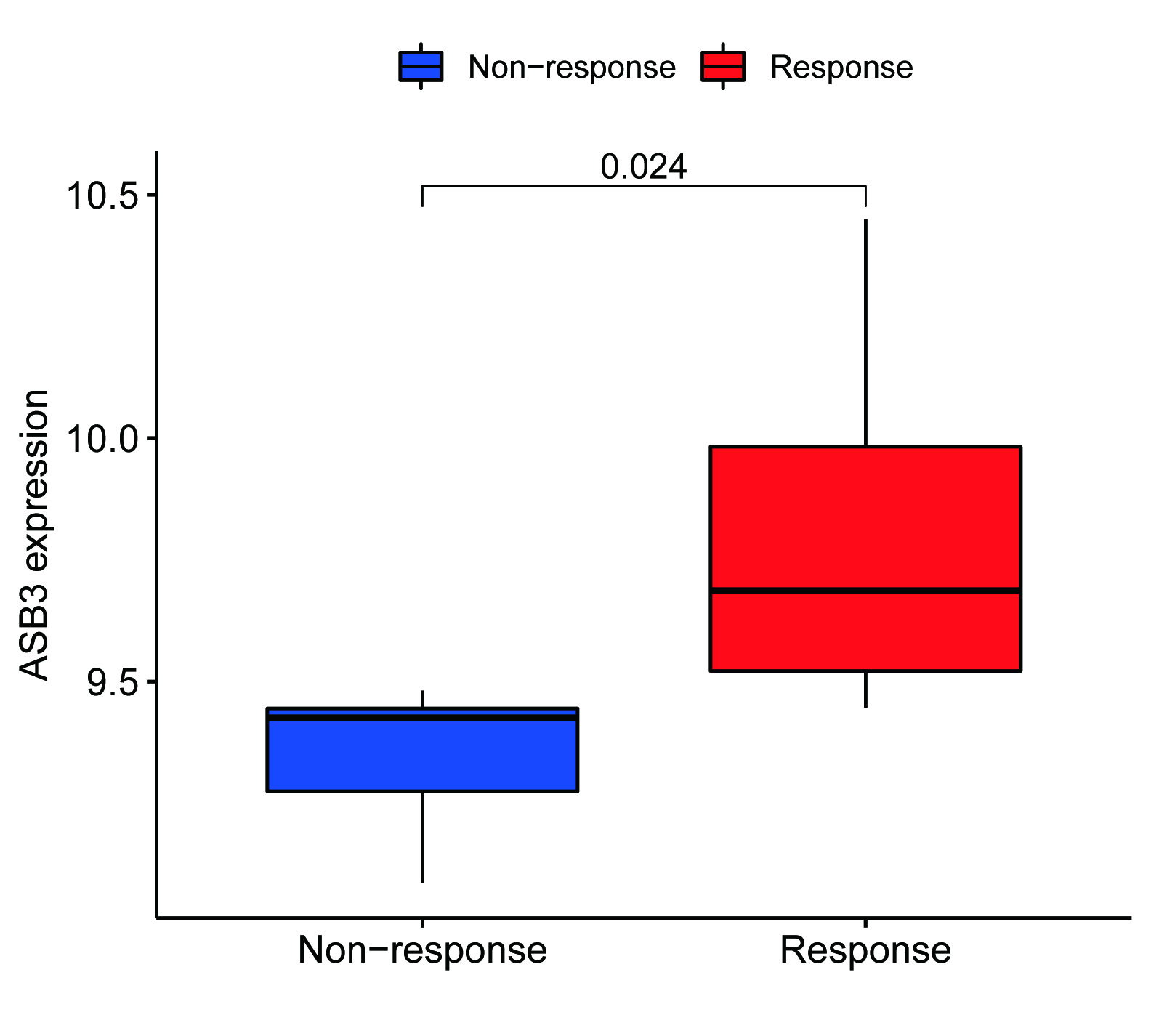

Supplement: Supplementary Figure 5 — Enrichment plots from GSEA GO (Independent cohort). (A) B Cell Mediated Immunity. (B) Cytokine Activity. (C) Immunoglobulin Receptor Binding. (D) Humoral Immune Response Mediated by Circulating Immunoglobulin. (E) Antigen Binding. (F) Complement Activation. [file Image_5.tif]

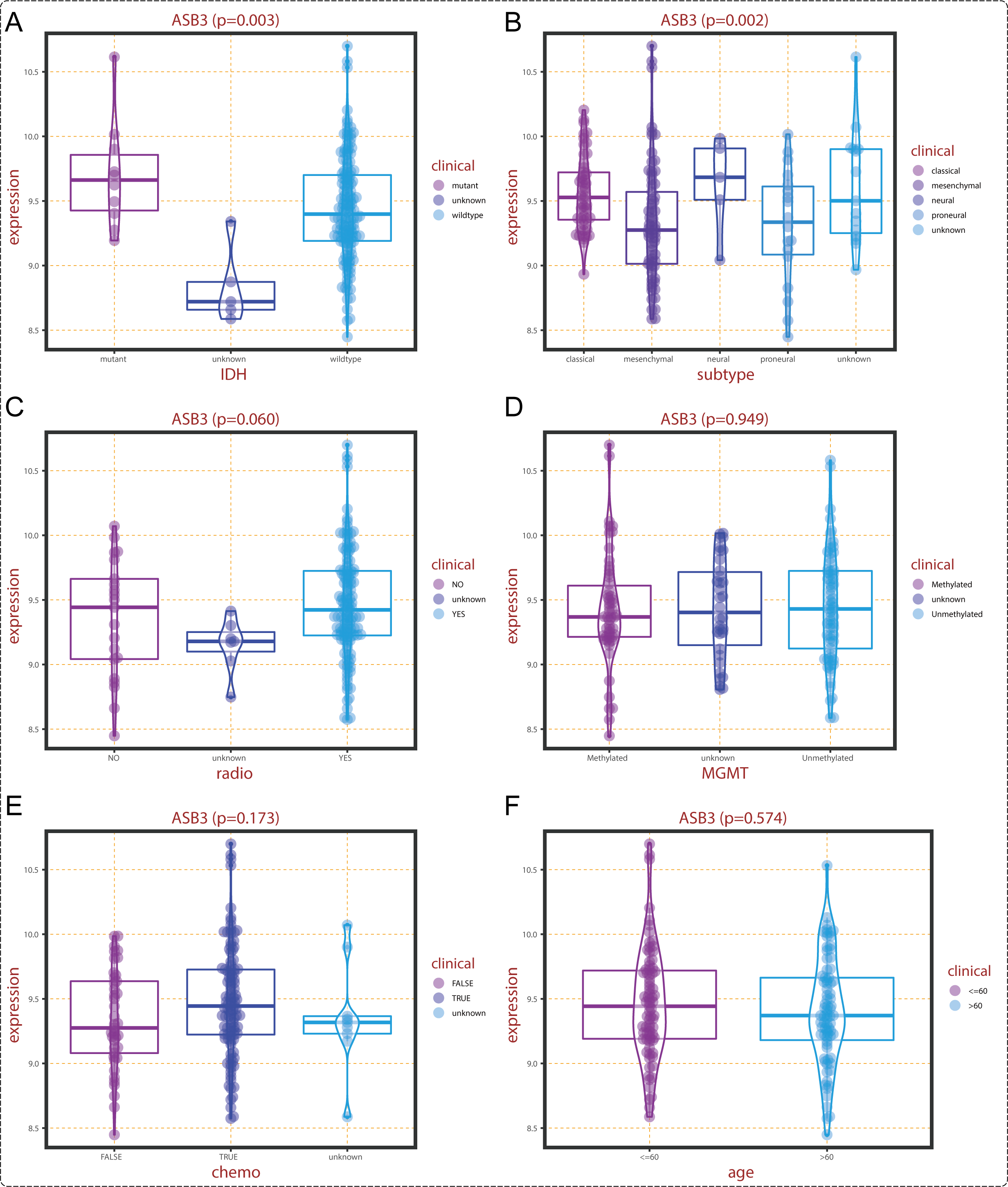

Supplement: Supplementary Figure 6 — Enrichment plots from GSEA KEGG (Independent cohort). (A) Toll Like Receptor Signaling Pathway. (B) Lysosome. (C) Chemokine Signaling Pathway. (D) Cytokine-Cytokine Receptor Interaction. (E) Cell Adhesion Molecules CAMS. (F) Primary Immunodeficiency. [file Image_6.tif]

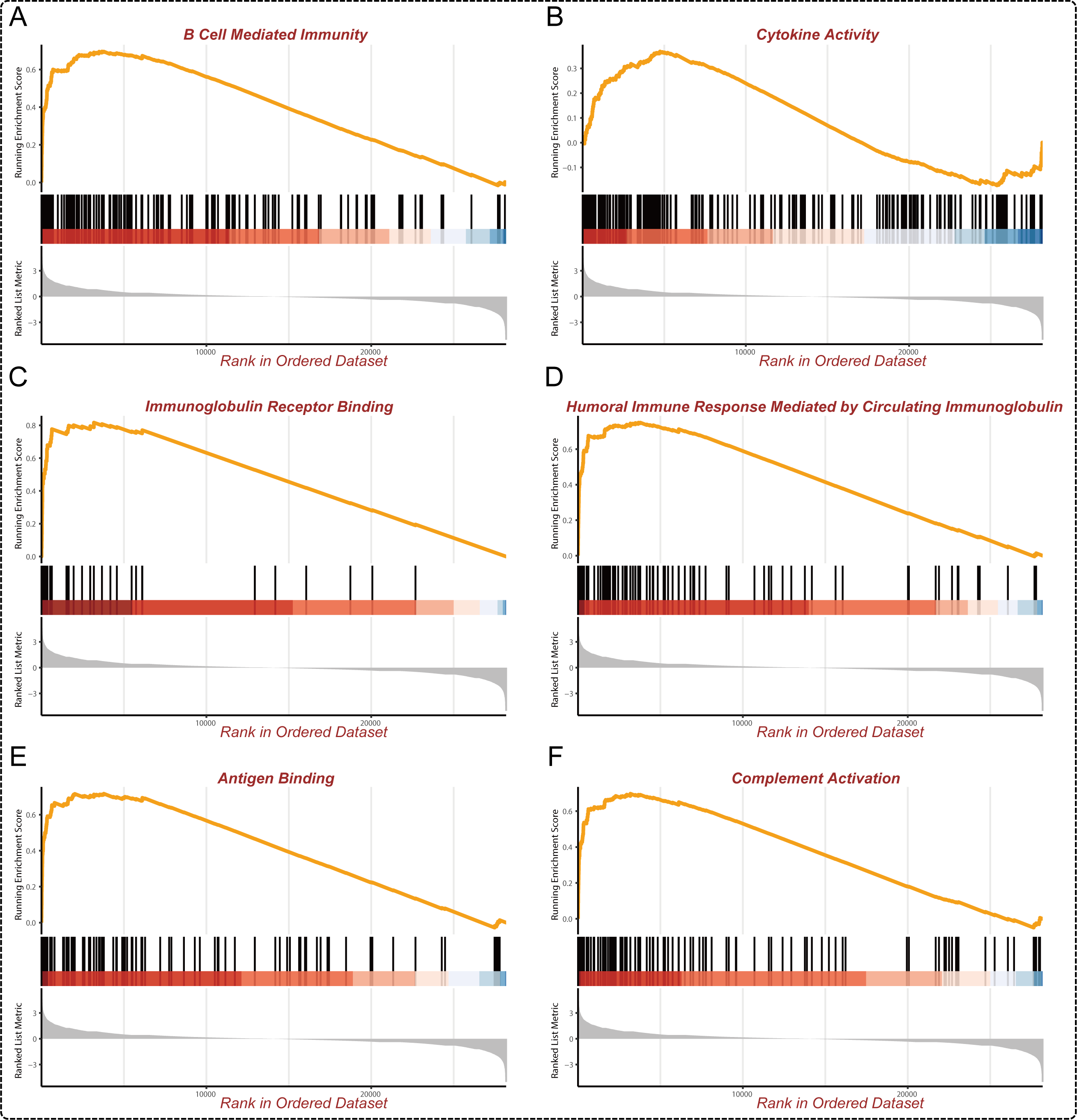

Supplement: Supplementary Figure 7 — Correlation of ASB3 expression and immunotherapy response in human renal cell carcinoma. [file Image_7.tif]
